# Defective Repair of Oxidative Base Lesions by the DNA Glycosylase Nth1 Associates with Multiple Telomere Defects

**DOI:** 10.1371/journal.pgen.1003639

**Published:** 2013-07-18

**Authors:** Haritha Vallabhaneni, Nathan O'Callaghan, Julia Sidorova, Yie Liu

**Affiliations:** 1 Laboratory of Molecular Gerontology, National Institute on Aging, National Institutes of Health, Baltimore, Maryland, United States of America; 2 Animal, Food and Health Sciences, CSIRO, Adelaide, Australia; 3 Department of Pathology, University of Washington, Seattle, Washington, United States of America; Chinese Academy of Sciences, China

## Abstract

Telomeres are chromosome end structures and are essential for maintenance of genome stability. Highly repetitive telomere sequences appear to be susceptible to oxidative stress-induced damage. Oxidation may therefore have a severe impact on telomere integrity and function. A wide spectrum of oxidative pyrimidine-derivatives has been reported, including thymine glycol (Tg), that are primarily removed by a DNA glycosylase, Endonuclease III-like protein 1 (Nth1). Here, we investigate the effect of Nth1 deficiency on telomere integrity in mice. *Nth1* null (*Nth1^−/−^*) mouse tissues and primary MEFs harbor higher levels of Endonuclease III-sensitive DNA lesions at telomeric repeats, in comparison to a non-telomeric locus. Furthermore, oxidative DNA damage induced by acute exposure to an oxidant is repaired slowly at telomeres in *Nth1^−/−^* MEFs. Although telomere length is not affected in the hematopoietic tissues of *Nth1^−/−^* adult mice, telomeres suffer from attrition and increased recombination and DNA damage foci formation in *Nth1^−/−^* bone marrow cells that are stimulated *ex vivo* in the presence of 20% oxygen. Nth1 deficiency also enhances telomere fragility in mice. Lastly, in a telomerase null background, *Nth1^−/−^* bone marrow cells undergo severe telomere loss at some chromosome ends and cell apoptosis upon replicative stress. These results suggest that Nth1 plays an important role in telomere maintenance and base repair against oxidative stress-induced base modifications. The fact that telomerase deficiency can exacerbate telomere shortening in *Nth1* deficient mouse cells supports that base excision repair cooperates with telomerase to maintain telomere integrity.

## Author Summary

Oxidative stress causes DNA base damage that is mainly repaired by base excision repair pathway, where a DNA glycosylase initiates the recognition and removal of specific base damage. Mammalian telomeres are composed of repetitive purine and pyrimidine bases, TTAGGG, which are prone to damage by oxidation. Though previously we have shown that oxidative damage to a purine base, guanine (G), affects telomere integrity, damage to other telomere bases, *e.g.* a pyrimidine base, thymine (T), may also occur and potentially disrupt telomere maintenance. In order to test this hypothesis, we utilize a mouse model lacking Endonuclease III-like protein 1 (Nth1), a DNA glycosylase that primarily recognizes and excises oxidative thymine and other pyrimidine damage. We show that *Nth1* deficient mouse cells have higher levels of oxidative base damage at telomeres and display multiple telomere defects including telomere loss. Our studies support that besides oxidative guanine damage, other oxidative base damage can interfere with telomere maintenance. These results may be relevant to understanding how oxidative base damage and inefficient DNA repair contribute to telomere loss, aging and cancer susceptibility in humans and other mammals.

## Introduction

All eukaryotic linear chromosome ends consist of complex nucleoprotein structures, called telomeres. Telomeres are composed of tandem repeat sequences 5′-(TTAGGG)_n_-3′ whose lengths vary from about 10 kbps in humans to up to 100 kbps in mice. In mammals, telomere DNA is bound by the shelterin complex, including telomere repeat binding proteins TRF1, TRF2, and POT1 [1]. Telomeres prevent recombinogenic chromosome ends from inducing chromosomal rearrangements that destabilize the eukaryotic genome. Telomere attrition (shortening) or other forms of telomere dysfunction can evoke an ATM- or ATR- dependent DNA damage response that results in 53BP1 and γ-H2AX foci formation at telomeres, Chk1 and Chk2 phosphorylation, and the induction of cell cycle arrest, senescence, or apoptosis [2].

Telomere maintenance involves telomerase extension and telomere recombination, replication, and capping [3]. It is also affected by other factors, the most notable being oxidative stress [4]. Telomere length decreases after each cell division, due to the inability of DNA polymerases to completely replicate DNA ends. However, telomerase counteracts telomere shortening by replenishing telomeric repeats. Telomerase is a ribonucleoprotein complex composed of telomerase reverse transcriptase (*Tert*) and an RNA component (*Terc*) [3]. In telomerase null mice, telomere length gradually decreases to a critical length, which activates a DNA damage checkpoint primarily in highly proliferating organs, such as bone marrow [5]. Recent data suggests that telomeres pose a challenge to replication machinery, resulting in defects similar to aphidicolin-induced fragile sites possibly caused by replication fork stalling [6], [7]. It has been suggested that the shelterin component, TRF1, might recruit BLM or RTEL helicases to telomeres, thereby helping to resolve G-quadruplex structures that may inhibit telomere replication [7].

Telomeric DNA appears to be more susceptible to damage as a result of exposure to exogenous physical and chemical agents, such as oxidants [8]–[12]. For example, single stranded breaks (SSBs) and base damage preferentially occur in telomeres in human cells with oxidative stress [8], [9]. Furthermore, oxidant-induced telomeric DNA damage triggers a persistent DNA damage response [12]. Although it has been proposed that G-rich telomeric sequence may be more susceptible to oxidative DNA damage [13]–[15], a wide spectrum of oxidized pyrimidine-derivatives, *e. g.* 5-hydroxycytosine (5-OH-Cyt), 5-hydroxyuracil (5-OH-Ura), and Tg has also been reported [16], [17] and may exist at telomeres. For example, thymine is relatively rich in telomere repeats and could be modified into Tg by oxidation, and Tg might potentially hamper DNA replication [18]–[20]. Furthermore, oxidative base lesions in telomere substrates reduce the binding of telomere binding proteins to telomere DNA [21], [22], which may, in turn, affect telomere maintenance.

Non-bulky oxidative base lesions are primarily repaired by the base excision repair (BER) pathway, and the first step in BER is carried out by a DNA glycosylase, which recognizes and removes damaged bases [23]. Mammalian cells express several glycosylases with overlapping but distinct specificity for various base lesions [23]. For example, 8-oxoguanine DNA glycosylase 1 (Ogg1) mostly recognizes oxidized guanine lesions, e.g. 8-oxoG, while Nth1 primarily recognizes oxidized bases other than 8-oxoG, e.g. 5-OH-Cyt, 5-OH-Ura and Tg [23], [24]. Nth1 is highly expressed during early and mid-S phase, suggesting that it plays a role in replicative repair [25]. Ogg1 deficiency results in the accumulation of oxidative 8-oxoG lesions in telomeres and attenuates telomere integrity [22], [26]. However, it is unclear if other types of oxidative base lesions might accumulate at telomeres and if ablation of their repair could affect telomere maintenance. Here, we utilize *Nth1* null mice to evaluate these probabilities.

## Results

### Elevated level of Endonuclease III-sensitive DNA lesions at telomeres in Nth1 deficient mouse tissues and primary MEFs

To determine if oxidative base lesions accumulate at telomeres, genomic DNA was isolated from wild-type and *Nth1^−/−^* mouse kidneys and primary MEFs, treated with Endonuclease III, and measured for Endonuclease III-sensitive lesions at telomeres using a quantitative telomere PCR method [27]. *E. coli* Endonuclease III has similar substrate specificity profiles as mammalian Nth1 and primarily excises oxidized bases including 5-OH-Cyt, 5-OH-Ura and Tg, resulting in abasic sites and subsequently single strand breaks (SSBs) [23] that impair PCR kinetics. The more base lesions are at telomeres, the more DNA nicks are generated by Endonuclease III treatment and hence the higher Ct values are produced. To eliminate interference by other potential DNA replication blocking lesions, *e.g.* spontaneous DNA strand breaks at telomeres, a duplicate mock digestion was set up for each corresponding sample in which Endonuclease III was excluded. Endonuclease III-sensitive lesions in a sample were normalized by comparing PCR kinetics in the mock- and Endonuclease III- treated samples, *i.e.* change in cycle threshold (ΔC_t_ = C_t_ treated - C_t_ mock) [27]. A standard curve for Endonuclease III-sensitive lesions was generated using synthetic telomere oligonucleotides containing various numbers of Tg lesions (Figure S1A and Table S1) and was used to calculate the relative numbers of Endonuclease III-sensitive lesions per kilobase of telomeric DNA in each sample as described by O'Callaghan *et al* [27]. As shown in Figure S1B, mock- and Endonuclease III-treated Tg-free telomere oligonucleotides yield comparable Ct values. In contrast, Endonuclease III-treated Tg-containing telomere oligonucleotides show higher Ct values in comparison to mock-treated Tg-containing telomere oligonucleotides (Figure S1B). Similarly, mock-treated genomic DNA from wild-type and *Nth1^−/−^* mice have comparable Ct values, while Endonuclease III-treated *Nth1^−/−^* mouse genomic DNA displays higher Ct values than Endonuclease III-treated wild-type mouse genomic DNA (Figure S1C). These observations support that difference in PCR kinetics in mock- and Endonuclease III-treated mouse genomic DNA samples is due to cleavage of Endonuclease III-sensitive lesions in the DNA strand.

**Figure 1:**
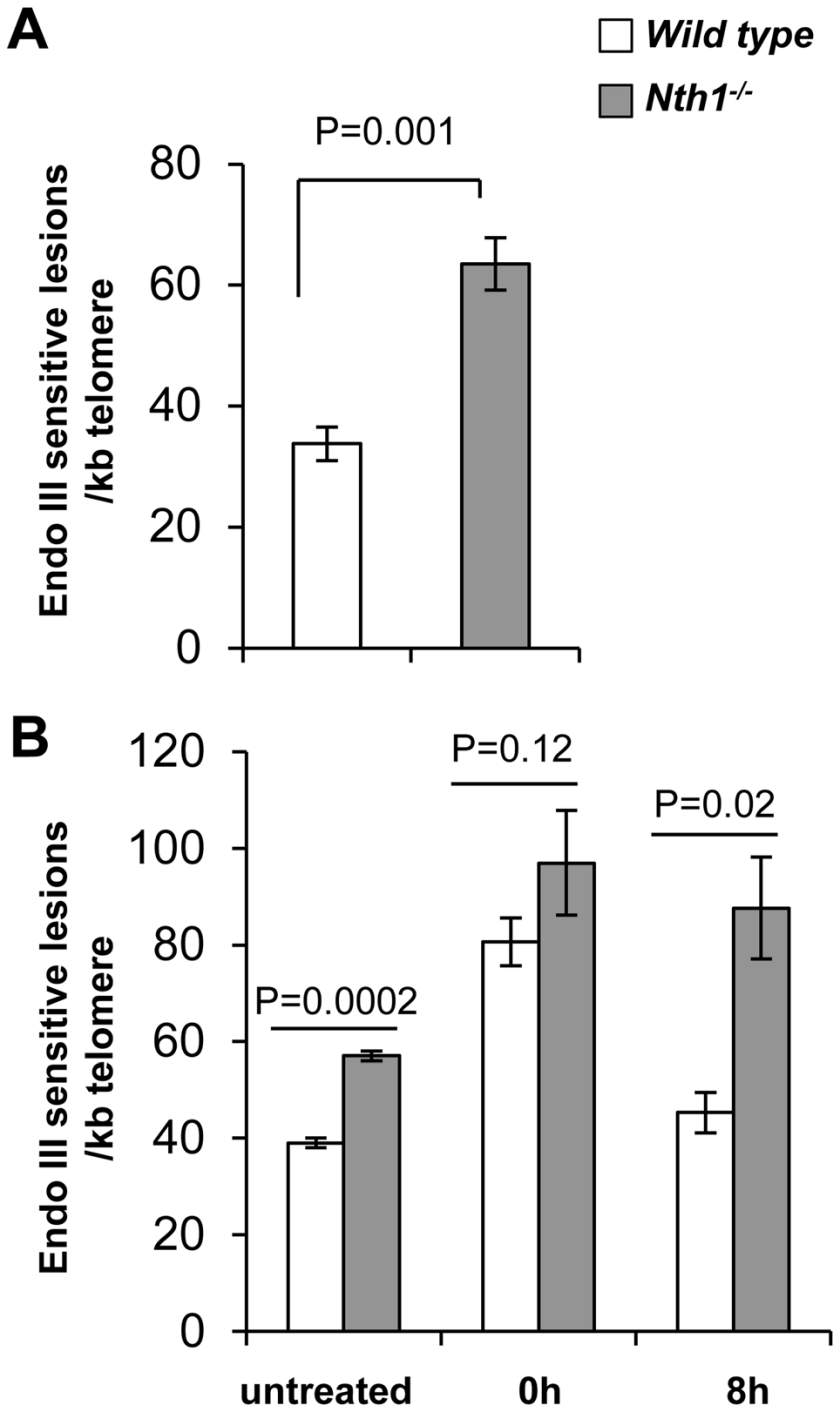
Detection of oxidative DNA base lesions at telomeres in mice. Genomic DNA is detected for Endonuclease III-sensitive DNA lesions per kilobase of telomeric DNA by a modified quantitative telomere PCR method. (A) Endonuclease III-sensitive DNA lesions in wild-type and *Nth1^−/−^* mouse kidney (n = 6 mice). (B) Endonuclease III-sensitive DNA lesions in primary MEFs (untreated) or MEFs exposed to 5 µM Benzo(a)pyrene for 24 hours followed by recovery for 0 hour or 8 hours. Each sample is analyzed in triplicate. Error bars denote standard deviation. P-values are calculated using a Student's *t*-test and adjusted using Benjamini-Hochberg False Discovery Rate-controlling method [57]. P-values<0.05 are statistically significant using the above method.

Genomic DNA from *Nth1^−/−^* kidney tissue and MEFs has about 2-fold and 1.8-fold more Endonuclease III-sensitive DNA lesions at telomeres, compared to that from wild type (Figure 1A and B). However, Endonuclease III-sensitive DNA lesions in an amplicon within a non-telomere locus (the *36B4* gene) are comparable in wild-type and *Nth1^−/−^* kidney tissue and MEFs, in contrast to those within telomeric repeats (Figure S2). Thus, *Nth1* deficient mouse cells harbor a higher density of Endonuclease III-sensitive DNA lesions at telomeres. These results are consistent with previous reports that oxidative 8-oxoG lesions and single strand breaks preferentially accumulate at telomeres in mammalian cells [8], [9].

To determine if ablation of Nth1 function affects oxidative base repair kinetics at telomeres, wild-type or *Nth1^−/−^* primary MEFs were treated with an oxidant, benzo[a]pyrene that induces oxidative modifications at DNA [28]. Primary MEFs were exposed to 5 µM benzo(a)pyrene for 24 hours and then recovered for 8 hours. Immediately after exposure (0 hour), an approximately 2-fold increase in the number of Endonuclease III-sensitive DNA lesions is detected at telomeres in wild-type and *Nth1^−/−^* MEFs, compared to that in untreated MEFs. Persistent Endonuclease III-sensitive DNA lesions are detected at telomeres during the recovery period in *Nth1^−/−^* MEFs, while they return to the basal levels in wild-type MEFs within 8 hours after exposure is terminated (Figure 1B). These data suggest that Nth1 plays an important role in repairing Endonuclease III-sensitive DNA lesions in telomeres *in vivo*.

### Higher incidence of fragile telomeres in Nth1 deficient mouse cells

**Figure 2:**
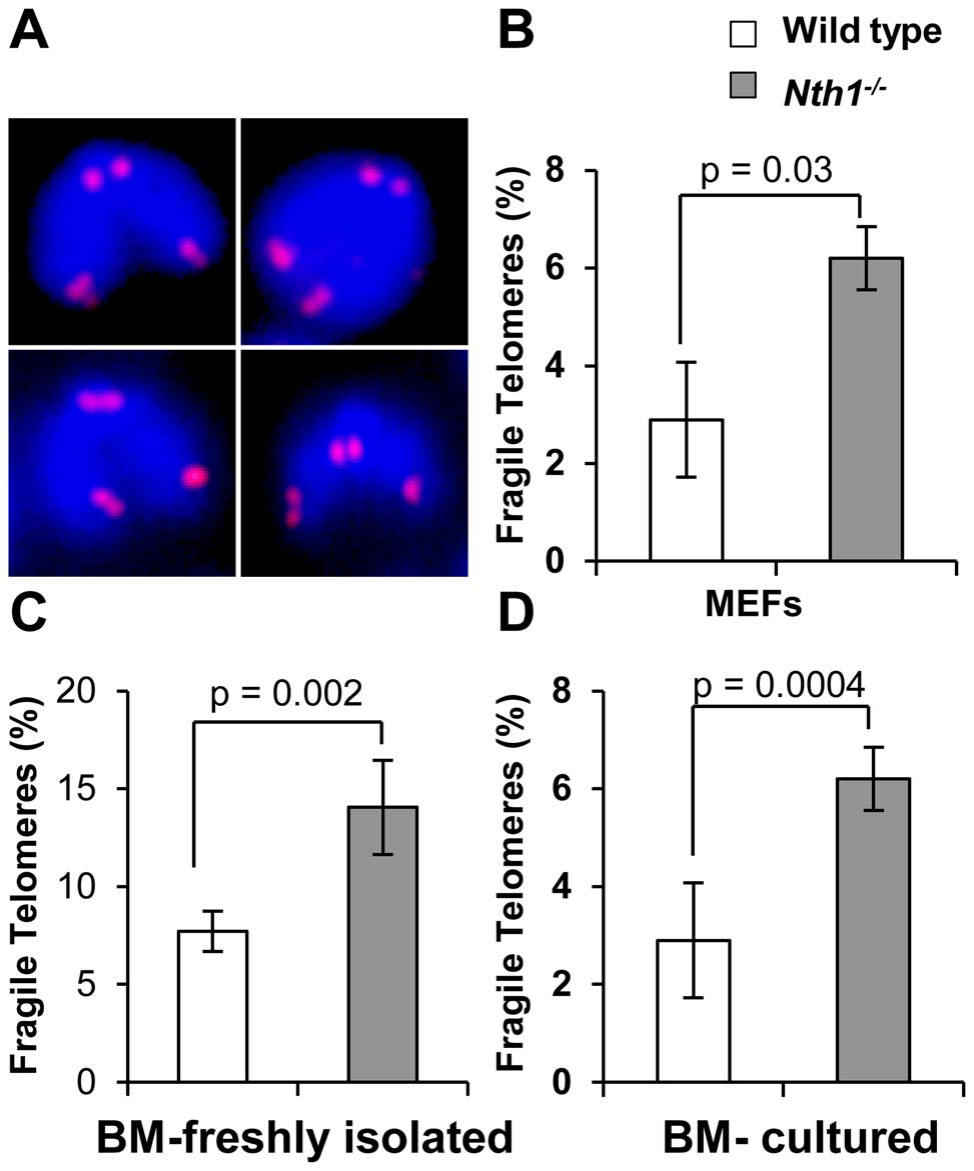
Fragile telomeres in wild-type and *Nth1^−/−^* mouse cells. (A) Examples of fragile telomeres by telomere-FISH analysis. (B–D) Percentage of fragile telomeres in primary MEFs (4 MEF lineages), freshly isolated bone marrow cells (11 mice), and bone marrow cells that are stimulated in culture (10 mice). At least 60 metaphases/sample are counted. Error bars indicate standard deviation. P-values are calculated using a Student's *t*-test.

Defective telomere replication associates with aberrant telomeres characterized by split signals or multiple signals at a telomere [7] (Figure 2A). These aberrant structures are described as fragile telomeres and are found in cells treated with low doses of aphidicolin (a specific inhibitor of DNA polymerases) and in cells deficient in the shelterin proteins and/or other proteins required for telomere replication [7], [29]–[33]. Oxidized bases might impede DNA replication [18]–[20] and perturb telomere-bound TRF1 [21] thereby disrupting telomere replication. Consistent with the hypothesis, the incidence of fragile telomeres is found to be higher in *Nth1^−/−^* primary MEFs and bone marrow cells than that in wild-type controls (Figure 2B–2D). Fragile telomeres are also evident in *Ogg1^−/−^* mouse bone marrow cells (Figure S3). Lastly, low-dose aphidicolin treatment enhances the numbers of fragile telomeres in wild-type cells, but not significantly in *Nth1^−/−^* cells (Table 1), supporting the idea that low doses of aphidicolin and Nth1 deficiency might influence telomere replication and hence fragility via the same mechanism. Collectively, these results suggest that oxidized bases and/or Nth1 deficiency affect telomere replication.

**Table 1:** Quantification of fragile telomeres in wild-type and *Nth1^−/−^* mouse bone marrow cells and primary MEFs treated with a low dose of aphidicolin.

	Fragile telomeres (%)	
**Bone marrow**	**Untreated**	**Aphidicolin (0.2 µM)**
Wild type	2.91±0.48	6.23±1.1* ^P = 0.02^
*Nth1^−/−^*	4.62±0.58* ^P = 0.0004^	4.16±1.5
**Primary MEFs**	**Untreated**	**Aphidicolin (0.2 µM)**
Wild type	3.4±1.1	8.36±0.28* ^P = 0.03^
*Nth1^−/−^*	6.2±0.65* ^P = 0.02^	6.82±2.5

Values indicate percentage of fragile telomeres. Bone marrow cells (6 mice) and MEFs (4 lines) are treated with aphidicolin for 16 hours. At least 50 metaphases are counted.

* denotes P-values that yield significant difference compared with wild type (untreated).

### Higher incidence of genomic and telomeric DNA damage foci in Nth1 deficient mouse cells

**Figure 3:**
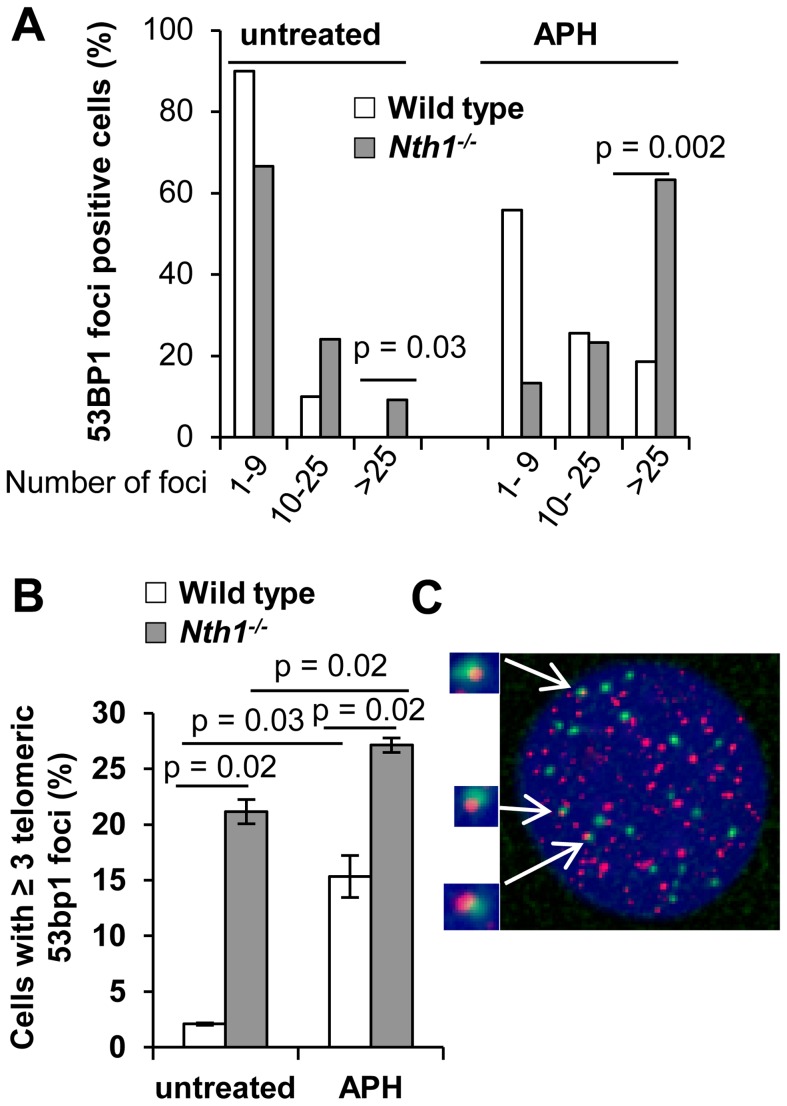
DNA damage foci in wild-type and *Nth1^−/−^* primary MEFs. 53BP1 foci at genome or telomeres are detected by IF and IF-telomere FISH, respectively. (A) Percentage of wild-type and *Nth1^−/−^* cells with various numbers of 53BP1 foci in the genome. (B) Percentage of wild-type and *Nth1^−/−^* cells with greater than or equal to three 53BP1 foci that colocalize with telomere DNA. APH: cells treated with 0.2 uM aphidicolin for 16 hours. (C) A representative *Nth1^−/−^* cell showing telomeric DNA (red) and 53BP1 foci (green). N = 4 mice, at least 100 cells/sample are counted. Error bars indicate standard deviation. P-values are calculated using a Student's *t*-test and adjusted using Benjamini-Hochberg False Discovery Rate -controlling method. P-values<0.05 are statistically significant using the above method.

Persistent oxidative base lesions may cause stalling of DNA replication leading to DNA damage signaling and double strand breaks (DSBs) at telomeres [34]. Oxidative base lesions may also inhibit the binding of telomere binding proteins to telomeric DNA [21] and ultimately their ability to mask telomeres from triggering a DNA damage response [2]. 53BP1 foci formation, a marker for DSBs or DNA damage signal [2], [35], was therefore examined to assess DSBs or a DNA damage response in the genome and telomeres of wild-type and *Nth1^−/−^* primary MEFs. The distribution of cells with different numbers of total 53BP1 foci were measured by indirect immunofluorescence (IF), and telomeric 53BP1 foci were then identified by IF-telomere FISH. A greater fraction of *Nth1^−/−^* MEFs has 10–25 or >25 53BP1 foci (24% and 10%, respectively) in comparison to wild-type MEFs (10% and 0%, respectively) (Figure 3A). In addition, more *Nth1^−/−^* MEFs have ≥3 telomeric 53BP1 foci in comparison to wild-type MEFs (21% and 2%, respectively) (Figure 3B). These results demonstrate that ablation of Nth1 function causes genomic and telomeric damage foci formation. Similar results were obtained in the wild-type and *Nth1^−/−^* bone marrow cells that were stimulated in culture (Figure S4).

To further explore the relationship between DNA damage foci formation and DNA replication, wild-type and *Nth1^−/−^* primary MEFs were cultured in the presence of a low dose of aphidicolin (0.2 µM). After exposure to aphidicolin for 16 hours, the fraction of wild-type MEFs with >25 53BP1 foci is significantly increased (0% versus 18%; untreated versus treated) (Figure 3A). Aphidicolin treatment also increases the fraction of wild-type cells with ≥3 telomeric 53BP1 foci (2% versus 15%, untreated versus treated) (Figure 3B). Conversely, replication stress only moderately enhances the fraction of *Nth1^−/−^* cells with >25 53BP1 foci (24% versus 63%, untreated versus treated) (Figure 3A) and the fraction of *Nth1^−/−^* cells with ≥3 telomeric 53BP1 foci (20% versus 27%, untreated versus treated) (Figure 3B). Hence, replication stress significantly enhances telomere DNA damage in wild-type cells, but, to a lesser extent in *Nth1^−/−^* cells, supporting our hypothesis that Nth1 deficiency and low doses of aphidicolin may cause similar replication defects, thereby contributing to DNA damage foci formation in the genome and the telomeres.

Persistent DNA damage may trigger phosphorylation of DNA damage checkpoint effector proteins, Chk1 and Chk2 [35]. Because *Nth1^−/−^* primary MEFs display an increase in genomic and telomeric 53BP1 foci, we examined *Nth1^−/−^* primary MEFs for the presence of phosphorylated forms of Chk1 and Chk2 by Western blot analysis. Despite the presence of DNA damage foci, Chk1 and Chk2 phosphorylation is not detected in *Nth1^−/−^* primary MEFs (Figure S5). High-dose aphidicolin (5 µM) and gamma irradiation (10 Gy) induce DSBs and Chk1 and Chk2 phosphorylation in mammalian cells [36], [37], and these treatments cause Chk1 and Chk2 phosphorylation in *Nth1* ^−/−^ primary MEFs (Figure S5). Thus, the DNA damage check point response is intact in *Nth1* ^−/−^ primary MEFs. Collectively, these results suggest that the levels of DNA damage in *Nth1^−/−^* mouse cells might not be high enough to evoke a persistent DNA damage response.

### Nth1 is involved in telomere length maintenance in mice

As shown above, Nth1 deficiency leads to telomere fragility and telomere DNA damage. These defects may affect telomere length maintenance or distribution. We therefore examined telomere length in wild-type and *Nth1^−/−^* mice by telomere-FISH. Flow-FISH analysis reveals that average telomere length does not undergo a significant change in *Nth1^−/−^* hematopoietic tissues, *i.e.* bone marrow and spleen (Figure S6A). Similar results are obtained by Q-FISH analysis of metaphase spreads of freshly isolated wild-type and *Nth1^−/−^* bone marrow cells (Figure S6B). It is noteworthy that marginal fluctuation in telomere length (lengthening or shortening) is occasionally observed in *Nth1^−/−^* hematopoietic tissues (data not shown).

**Figure 4:**
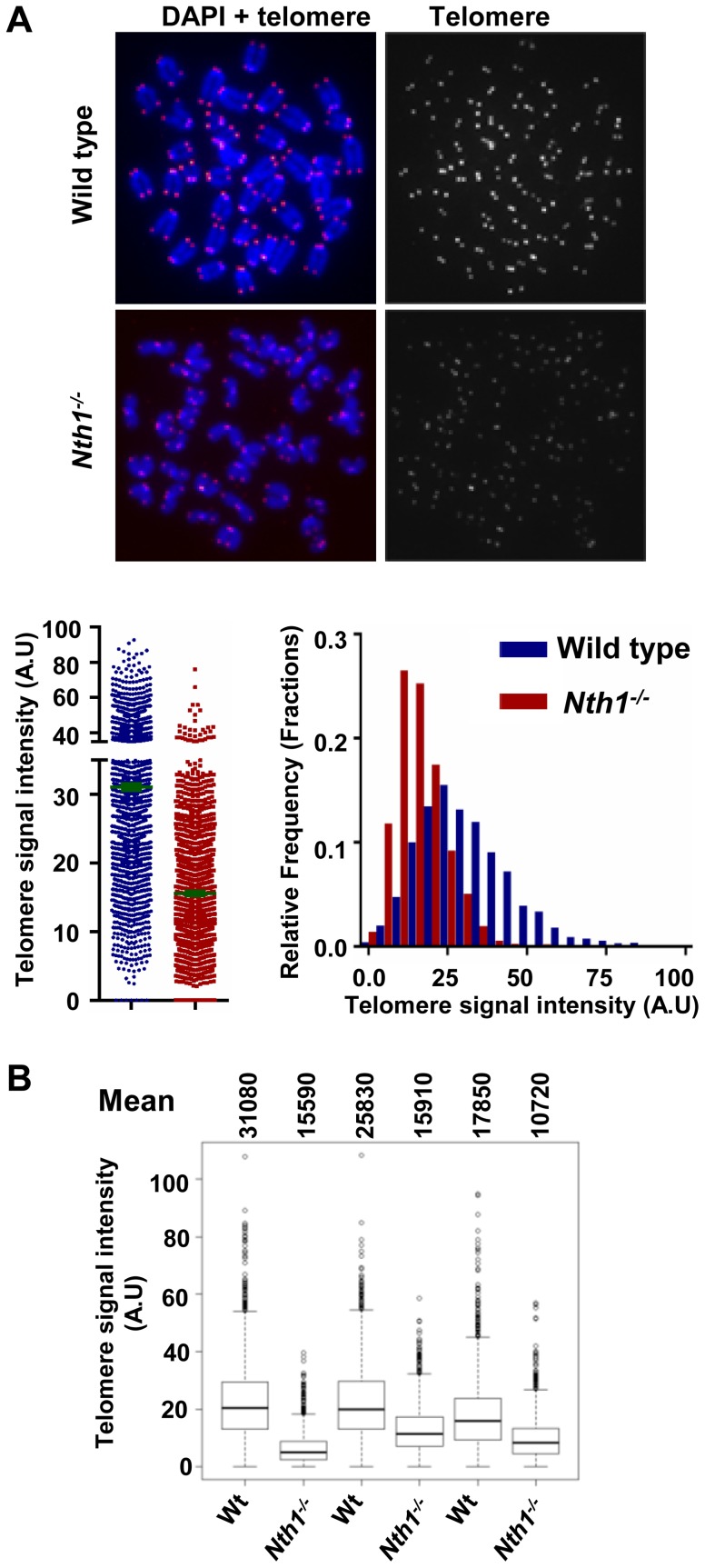
Telomere length in wild-type and *Nth1^−/−^* mouse cells. Q-FISH analysis of *ex vivo* stimulated bone marrow cells. (A) Representative metaphase spreads of wild-type and *Nth1^−/−^* mouse bone marrow cells showing DAPI staining (blue) and telomere fluorescence signals (red dots in left panel and white dots in right panel). Quantitative measurement of telomere signal intensity is shown in a jitter plot displaying complete distribution of telomeres with diverse signal intensity (left panel) and in a combined histogram displaying relative frequency of telomeres plotted against telomere signal intensity (right panel). Bars (in green) denote mean telomere signal intensity. (B) Box plot of telomere signal intensity of bone marrow cells from three pairs of wild-type and *Nth1^−/−^* mice. Mean values are shown above each mouse sample. Telomere signal intensity is depicted in arbitrary units (A.U).

We also investigated if an increase in cell proliferation or oxidative stress could exacerbate telomere defects in *Nth1^−/−^* hematopoietic cells. Mouse bone marrow cells were stimulated by interleukin 6 and stem cell factor in culture in the presence of 20% oxygen. Under these conditions, *Nth1^−/−^* cells show reduced telomere signal intensity compared to wild-type cells (Figure 4). Thus, replication and/or oxidative stress can induce telomere attrition in the absence of Nth1.

### Nth1 and telomerase cooperate in telomere maintenance

**Figure 5:**
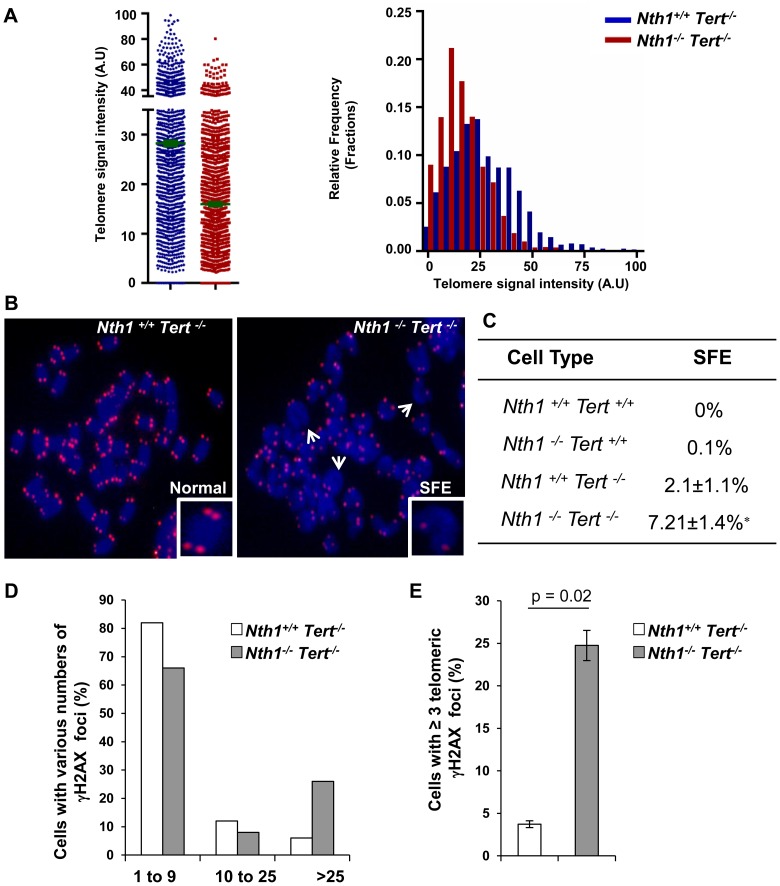
Telomere length and DNA damage foci in *Nth1^+/+^Tert^−/−^* and *Nth1^−/−^Tert^−/−^* mouse cells. (A) Q-FISH analysis of bone marrow cells derived from *Nth1^+/+^ Tert^−/−^* and *Nth1^−/−^Tert^−/−^* mice (n = 8). Representative quantitative measurement of telomere signal intensity is shown in jitter plot displaying complete distribution of telomeres with diverse signal intensity (left panel) and in a combined histogram displaying relative frequency of telomeres plotted against telomere signal intensity (right panel). Bars (in green) denote mean telomere signal intensity. (B) Representative metaphase spreads from indicated genotypes, showing enlarged chromosome ends with or without telomere signals (Normal and SFE, respectively). Arrows depict SFEs. (C) Quantification of SFEs in bone marrow cells with indicated genotypes (8 mice). At least 50 metaphases/sample are counted. Values depict mean values ± SD from each sample. P-values are calculated using a Student's *t*-test. * represents P = 0.01. (D–E) Percentage of *Nth1^+/+^ Tert^−/−^* and *Nth1^−/−^Tert^−/−^* cells with various numbers of γ-H2AX foci in the genome and telomeres by IF and IF-telomere FISH analysis, respectively. At least 100 cells/sample are counted. Error bars indicate standard deviation.

Telomerase is primarily responsible for telomere lengthening in *Ogg1* deficient *S. cerevisiae* [22]. While most human somatic and primary cells express a low or an undetectable level of telomerase [38]–[40], mouse cells from most laboratory strains constitutively express a high level of telomerase [41]. As a consequence, the impact of Nth1 deficiency on telomere integrity could be masked by telomerase in mouse cells. To explore this possibility, *Nth1* knockout mice were crossed into a strain lacking the telomerase reverse transcriptase, Tert to generate *Nth1* ^+/+^ *Tert* ^−/−^ and *Nth1* ^−/−^ *Tert* ^−/−^ mice. The bone marrow cells from *Nth1* ^+/+^ *Tert* ^−/−^ and *Nth1* ^−/−^ *Tert* ^−/−^ mice were examined for telomere length and DNA damage foci by Q-FISH and IF-telomere FISH, respectively. *Nth1* ^−/−^ *Tert* ^−/−^ mice have shorter telomere length than *Nth1* ^+/+^ *Tert* ^−/−^ mice (Figure 5A). Both *Nth1* ^+/+^ *Tert* ^−/−^ and *Nth1* ^−/−^ *Tert* ^−/−^ mice have detectable telomere signal free ends (SFEs), but remarkably, SFEs are significantly increased in *Nth1* ^−/−^ *Tert* ^−/−^ mice (7.21±1.4%) in comparison to *Nth1* ^+/+^ *Tert* ^−/−^ mice (2.1±1.1%), a phenotype that is not observed in the telomerase proficient background (Figure 5B and 5C). Furthermore, there are more genomic and telomeric γ-H2AX foci in *Nth1* ^−/−^ *Tert* ^−/−^ mice, compared to *Nth1* ^+/+^ *Tert* ^−/−^ mice (Figure 5D and 5E). These results provide evidence that telomerase deficiency exacerbates telomere defects in *Nth1* deficient cells.

Critically short telomeres can evoke a DNA damage response and result in cell apoptosis in highly proliferative organs [5], [42], [43]. We therefore examined for the presence of phosphorylated forms of Chk1 and Chk2 in *Nth1* ^+/+^ *Tert* ^−/−^ and *Nth1* ^−/−^ *Tert* ^−/−^ mouse cells by Western blot analysis (Figure S7A). *Nth1* ^+/+^ *Tert* ^−/−^ and *Nth1* ^−/−^ *Tert* ^−/−^ mouse cells display Chk1 and Chk2 phosphorylation (Fig. S7A), indicating that critically short telomeres in these telomerase null mice induce a DNA damage response. Despite the presence of higher number of critically short telomeres, the frequency of apoptosis is not elevated in *Nth1* ^−/−^ *Tert* ^−/−^ bone marrow cells (Figure S7B). Thus, the numbers of critically short telomeres have not reached its critical mass to influence cell viability in *Nth1* ^−/−^ *Tert* ^−/−^ mice. Although the basal level of apoptosis is comparable in *Nth1* ^+/+^ *Tert* ^−/−^ and *Nth1* ^−/−^ *Tert* ^−/−^ cells, a higher percentage of apoptosis is observed in *Nth1* ^−/−^ *Tert* ^−/−^ cells than in *Nth1* ^+/+^ *Tert* ^−/−^ cells after release from replication arrest induced by hydroxyurea (HU), an inhibitor of ribonucleotide reductase and therefore the synthesis of dNTP (Figure S7B, left panel). However, aphidicolin treatment causes similar increase in apoptosis in *Nth1* ^+/+^ *Tert* ^−/−^ and *Nth1* ^−/−^ *Tert* ^−/−^ cells (Figure S7B, right panel). Interestingly, HU treatment does not affect the rate of apoptotic cells in wild-type and *Nth1* ^−/−^ bone marrow cells in a telomerase proficient background (Figure S7C). Thus, HU, but not aphidicolin may induce apoptosis in *Nth1* ^−/−^ *Tert* ^−/−^ cells, likely by a mechanism involving a cooperative effect of telomerase and Nth1 against oxidative damage.

### Higher incidence of telomere sister chromatid exchange in Nth1 deficient mouse cells

**Figure 6:**
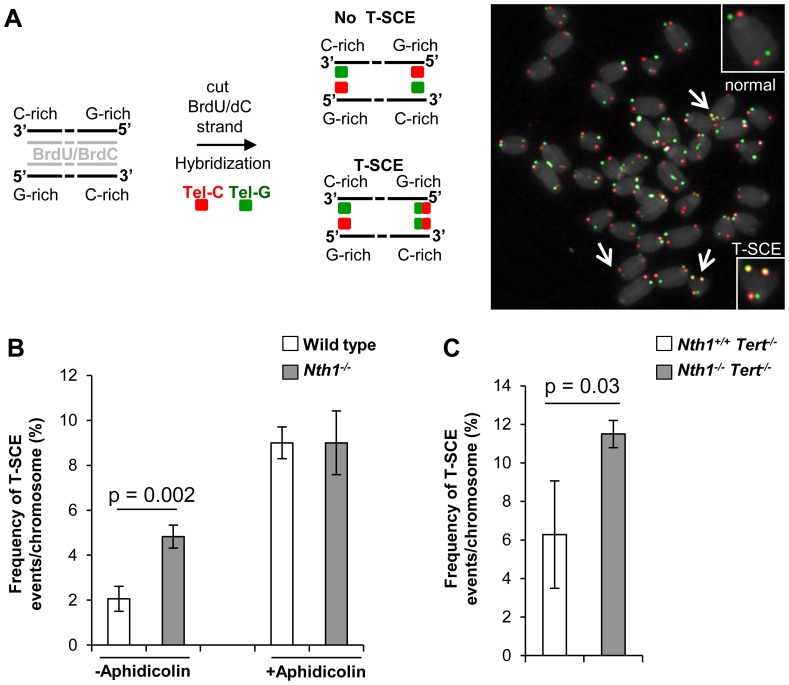
T-SCEs in wild-type and *Nth1^−/−^* mouse cells with or without telomerase. CO-FISH analysis of *ex vivo* stimulated bone marrow cells. (A) A schematic presentation of CO-FISH. In brief, newly synthesized strands with BrdU/BrdC are removed, leaving parental strands to be detected by Alexa488-labeled (TTAGGG)_3_ (in green) and Cy3-labeled telomere (CCCTAA)_3_ (in red) PNA probes. In an event of T-SCE, an end shows telomere signals in both green and red. An example of T-SCEs in a metaphase of *Nth1* ^−/−^ cell showing DAPI staining (gray), leading strand telomere fluorescence signals (red) and lagging strand telomere fluorescence signals (green). Arrows: T-SCEs. (B) T-SCE events in bone marrow cells of wild-type and *Nth1^−/−^* mice (n = 7) with (+) or without (−) aphidicolin treatment at the concentration of 0.2 µM for 16 hours. (C) T-SCE events in bone marrow cells of *Nth1^+/+^Tert^−/−^* and *Nth1^−/−^Tert^−/−^* mice (n = 4). At least 90 metaphases/sample are counted. Error bars indicate standard deviation. P-values are calculated using a Student's *t*-test.

Telomere sister chromatid exchange via homologous recombination, referred to as T-SCE, may be influenced by defective telomere maintenance or repair [44], [45]. In addition, oxidized bases may impede DNA replication [18]–[20] or interfere with binding of the shelterin proteins to telomeres [21], thereby inducing telomere recombination. We thus measured the frequency of T-SCEs in wild-type and *Nth1^−/−^* bone marrow cells by CO-FISH (Figure 6A). *Nth1^−/−^* cells display a higher rate of T-SCEs than wild-type cells (4.8 vs. 2.0% T-SCEs/chromosomes, respectively) (Figure 6B, left panel). Furthermore, in the telomerase deficient background a higher rate of T-SCEs is also observed in *Nth1^−/−^* mouse bone marrow cells (Figure 6C). Thus, Nth1 deficiency can induce telomere recombination, independently of telomerase. To explore the relationship between telomere recombination and DNA replication, wild-type and *Nth1^−/−^* bone marrow cells were treated with a low dose of aphidicolin for 16 hours. T-SCE events are significantly increased in wild-type mouse cells, but to a lesser extent, in *Nth1^−/−^* mouse cells (Figure 6B, right panel); supporting that Nth1 deficiency and low doses of aphidicolin may cause similar replication defects, thereby contributing to telomere recombination.

## Discussion

The BER pathway repairs non-bulky oxidative DNA lesions, including a number of potential replication-blocking lesions that hamper genome stability and cell viability, if left unrepaired. Telomeres are also critical for genomic integrity, because unprotected chromosome ends are highly prone to recombination and induce a DNA damage response. Telomeric DNA is sensitive to oxidative DNA damage [8], [9], [12], which may have negative impact on telomere maintenance. Here, we show that ablation of a BER repair protein, Nth1 leads to accumulation of telomere base damage and an increase in telomere attrition, fragility, recombination, and DNA damage foci. Telomerase deficiency exacerbates the telomere defects of *Nth1* deficient mice.

It has been shown that there is a tissue and age-dependent accumulation of 2,6.-diamino-4-hydroxy-5-formamidopyrimidine and 4, 6.-diamino-5-formamidopyrimidine in *Nth1* ^−/−^ mouse genome by gas and liquid chromatography/mass spectrometry detection methods [24], [46]. However, other Nth1-preferred base substrates, *e.g.* 5-OH-Cyt, 5-OH-Ura, and Tg do not persist in *Nth1* ^−/−^ mouse genome or are below the detection by the above methods [46]. In this study, we have utilized a quantitative PCR method to detect Endonuclease III (or Nth1)-sensitive DNA lesions at telomeric and non-telomeric loci in mice. A higher number of Endonuclease III-sensitive DNA lesions are detected at the telomeres, but not at the *36B4* locus in *Nth1* deficient kidney and MEFs, supporting that these oxidative base lesions preferentially accumulate at telomeres in the absence of Nth1. Since the extracts from *Nth1* ^−/−^ mice are unable to incise oxidized bases in 5-OH-Cyt, 5-OH-Ura, or Tg-containing oligonucleotides [47], it implies that there is a defective repair of these DNA base lesions in *Nth1* deficient mice, which could be exacerbated at telomeres. *Nth1* ^−/−^ MEFs are also inefficient in repairing telomeric Endonuclease III-sensitive DNA lesions that are induced by an oxidant, benzo[a]pyrene (Figure 1B). Our results support that Nth1 plays an important role in the removal of certain oxidative base lesions at telomeres. Similarly, *Ogg1* ^−/−^ mouse cells show elevated levels of Fpg-sensitive lesions and defective base repair at telomeres [26]. These results confirm the importance of BER proteins in protecting telomere DNA from oxidative damage.

Besides base lesions, 53BP1 and γ-H2AX foci are also detected in the telomeres of *Nth1* ^−/−^ mice. It is likely that these telomeric damage foci are derived from stalled replication forks. For example, the DNA polymerase inhibitor, aphidicolin, at low doses causes 7.5-fold increase in the percentage of wild-type cells positive for telomeric damage foci. *Nth1* ^−/−^ cells accumulate oxidized base lesions, which may also impede DNA polymerases, causing replication stress. The fact that low-dose aphidicolin treatment only causes 1.2-fold increase in the percentage of *Nth1* ^−/−^ cells positive for telomeric damage foci indicates that oxidative base lesions may interfere with telomere replication via a mechanism similar to that of low doses of aphidicolin. This trend is also observed for telomere fragility, telomere recombination and apoptosis in *Nth1* ^−/−^ cells that are treated with low doses of aphidicolin. We speculate that low doses of aphidicolin and Nth1 deficiency might influence these phenotypes by acting in the same pathway, such as inhibiting DNA polymerases. However, there is no detectable Chk1 activation in *Nth1* null cells, except with high-dose aphidicolin treatment. The differential response of *Nth1* ^−/−^ cells for telomere fragility and Chk1 activation could be due to different mechanisms and/or different doses (low or high) of aphidicolin in triggering these phenotypes. Sufficient DNA damage could trigger Chk1 activation [37], but the exact mechanism for the occurrence of fragile telomeres is yet unknown. Although Nth1 deficiency might sufficiently perturb DNA polymerases, to result in an increase in fragile telomeres, it may not lead to enough DNA damage to activate Chk1. Instead, high-dose aphidicolin treatment can significantly increase DNA damage [36], [37] and thus activate Chk1 in *Nth1* deficient cells.

Although oxidative base lesions may interfere with telomere replication, Nth1 could directly interact with and be involved in the progression of the replication apparatus [48], [49]. SSBs, which usually accumulate in telomeric DNA in the vicinity of oxidative base lesions [8], [13]–[15], could also be converted to DSBs during DNA replication [34] to interfere with telomere replication. Nevertheless, these potential defects fail to activate a persistent DNA damage response in *Nth1* ^−/−^ mice, as Chk1 and Chk2 are not phosphorylated in the null mice. This is consistent with the fact that deletion of *Nth1* alone does not lead to critically short telomeres and cell apoptosis.

When *Nth1* null mice are bred with *Tert* null mice, *Nth1* ^−/−^ *Tert* ^−/−^ mice harbor more critically short telomeres in comparison to *Nth1* ^+/+^ *Tert* ^−/−^ mice. Thus, Nth1 cooperates with telomerase to maintain telomere length. Interestingly, HU does not affect the rate of apoptosis in *Nth1* ^−/−^ mouse cells in a telomerase proficient background, but it promotes apoptosis in *Nth1* ^−/−^ mouse cells in a telomerase deficient background (Figure S7B). These results imply that HU induces apoptosis by a mechanism involving a cooperative effect of telomerase and Nth1 against oxidative damage. *Nth1* ^+/+^ *Tert* ^−/−^ and *Nth1* ^−/−^ *Tert* ^−/−^ mice are currently being propagated for additional generations to further exhaust telomere reserves, which may help reveal the impact of defective oxidative base lesion repair on telomere function and cell viability in mouse aging.

*Nth1* deficient mouse cells have a higher incidence of fragile telomeres, implying that replication encounters problems at telomeric DNA. We speculate that some Endonuclease III-sensitive base lesions, *e.g.* Tg pose a problem to the replication machinery in *Nth1* deficient mice. However, fragile telomeres are also increased in *Ogg1* ^−/−^ mice that harbor 8-oxoG. Thus, the presence of oxidative base lesions may nevertheless affect telomere replication, independently of the nature of the lesions. Oxidative base lesions may also impair telomere replication via perturbing the binding of TRF1 to telomeres [21], which is required for telomere replication [7]. The replication inhibitor aphidicolin does not increase the frequency of fragile telomeres in *Nth1* deficient mice. Similarly, aphidicolin fails to enhance fragile telomeres in mutant mice deficient in genes facilitating telomere replication, e.g. ATR or CTC1 [29], [31], [32]. These results further support our hypothesis that telomere replication is defective in *Nth1* deficient mice.

*Ex vivo*-stimulated *Nth1* deficient bone marrow cells display increased T-SCE events, which possibly reflects replication problem and DNA damage in telomeric DNA in *Nth1* null cells [45]. In line with this, low-dose aphidicolin treatment increases T-SCE events in wild-type, but not in *Nth1* deficient mouse cells (Figure 6B), supporting that low doses of aphidicolin or oxidative base lesion–induced replication stress contributes to telomere recombination. Alternatively, a DNA glycosylase may inhibit telomere recombination [50] and ablation of DNA glycosylase function would therefore relieve this inhibition and activate the recombination pathway. Lastly, Endonuclease III-sensitive base lesions may affect telomere recombination by disrupting the association of shelterin to telomeres [21].

Telomere length regulation involves many factors including telomerase, telomere binding proteins and telomere recombination/replication/capping. As discussed above, persistent oxidative DNA lesions and/or insufficient BER capacity could disrupt these processes, thereby interfering with telomere length homeostasis. Although *Nth1* deficient hematopoietic tissues have a normal distribution of telomere length, they undergo telomere shortening after being stimulated in culture with 20% oxygen. This suggests that the impact of Nth1 deficiency on telomere length is dependent on cell proliferation and/or exposure to higher oxygen levels. Cultured *Nth1* deficient bone marrow cells harbor more DNA damage that may also contribute to telomere attrition in these cells. Furthermore, damaged bases or Nth1 deficiency might interfere with telomere replication and thus telomere length, especially upon stimulating cell proliferation.

The *Nth1* deficient mouse model is a valuable tool for assessing the role of BER in telomere maintenance. However, somatic cells from *Nth1* deficient mice differ from human somatic cells in that the mouse cells express abundant telomerase activity. In this regard, it is important to note that the impact of Nth1 deficiency on telomere length is more severe in cells carrying the *Tert* null alleles. Moreover, *Nth1* and *Tert* double null mouse bone marrow cells demonstrate an elevated rate of apoptosis after replication stress. Thus, Nth1 and Tert might cooperate in maintaining telomere function in replicating cells. Because most human somatic cells express a low level of telomerase, age-dependent changes in BER capacity [51] could have a significant impact on telomere maintenance and cell viability in human tissues.

## Materials and Methods

### Mice and primary mouse cells

The generation of *Nth1* null (*Nth1^−/−^*) and *Tert* null (*Tert^−/−^*) mice was described previously [52], [53]. *Nth1^−/−^* mice were further backcrossed into C57BL/6 background. Wild-type and *Nth1^−/−^* mice were derived from heterozygous (*Nth1^+/−^*) breeders. To generate mice deficient for both *Nth1* and *Tert*, *Nth1* ^−/−^ mice was bred with *Tert* ^−/−^ mice to obtain *Nth1* ^+/−^ *Tert* ^+/−^, which were subsequently bred to generate *Nth1* ^+/+^ *Tert* ^−/−^ and *Nth1* ^−/−^ *Tert* ^−/−^ mice. Primary mouse embryonic fibroblasts (MEFs) were isolated from 13.5 day embryos of *Nth1^+/−^* female bred with *Nth1^+/−^* male and cultured in Dulbecco's Modified Eagle Medium containing 10% fetal bovine serum in CO_2_ incubator in the presence of 20% oxygen. Bone marrow cells were flushed from femurs and tibias and cultured in Iscove's modified Dulbecco's medium (Invitrogen) supplemented with 20% fetal calf serum with interleukin 6 (200 U/mL; Peprotech) and stem cell factor (100 ng/mL; Peprotech). Single cell suspensions of spleen were obtained by passing the spleen suspension through a cell strainer (70 µm, BD Falcon). All animal experiments were carried out according to the “Guide for the Care and Use of Laboratory Animals” (National Academy Press, USA, 1996), and were approved by the Institutional Animal Care and Use Committee of National Institute on Aging.

### Detection of oxidative base lesions in telomeres

Identification of oxidative base lesions in telomeres was performed as previously described [27]. In brief, DNA was isolated from mouse kidney and primary MEFs by salting out [22], [26]. To excise oxidative base lesions and generate strand breaks at the resulting abasic sites, DNA was digested with Endonuclease III. 400 ng of duplex oligomer or genomic DNA were incubated overnight with 12 units of Endonuclease III (New England Biolabs) in 1× NEB Endonuclease III buffer (20 mM Tris-HCl, 1 mM EDTA, 1 mM Dithiothreitol, pH 8.0). A duplicate digestion was also set up for each corresponding sample in mock digestion buffer (i.e. enzyme was excluded and substituted with H_2_O). All samples were set up on ice, then incubated at 37°C overnight to allow complete digestion and followed by the quantitative Real-Time amplification (qPCR) [54]. Five oligonucleotides containing TTAGGG repeats with 0, 1, 2, 4 or 8 thymine glycols were used for generation of a standard curve (Table S1). A reverse oligonucleotide was used to construct duplex substrates (GeneWorks, Adelaide). Forward and reverse oligonucleotides were mixed in a 1∶1 molar ratio. Annealing reactions were incubated at 95°C for 10 min and then cooled to room temperature for 30 min. For qPCR, each 20 µL reaction was composed as follows: 40 pg of digested or undigested oligonucleotides or 40 ng of digested or undigested genomic DNA, 1× SYBR Green master mix, 100 nM telo1 forward and 100 nM telo2 reverse primers [54]. All samples were run on an ABI 7300 Sequence Detection System with the SDS Ver. 1.9 software (Applied Biosystems). Cycling conditions were: 10 minutes at 95°C, followed by 40 cycles of 95°C for 15 seconds and 60°C for one minute. Each sample was analyzed in triplicate. PCR efficiencies and correlation coefficients for genomic DNA and synthetic oligonucleotides are shown in Table S2. The Ct values for all the samples were obtained from PCR reactions run under the same conditions using the same reagents, and the ΔCT value (C_t_ treated - C_t_ mock) for each sample were converted into numbers of Endonuclease III-sensitive lesions by comparison to the standard curve, as described previously [27].

### Telomere quantitative fluorescence in situ hybridization

Flow-FISH: The average telomere fluorescence in populations of splenocytes and bone marrow cells was measured according to a previously published protocol [55]. In each set, data were pooled from at least five individual mice. A telomere-specific FITC conjugated (CCCTAA)_3_ PNA probe (0.3 µg/mL, Panagene) was employed and telomere fluorescent signal intensity was measured by Accuri O6 flow cytometer using FlowJo software.

Q-FISH: Mice were injected with 100 µl of 0.5% colchicine intraperitoneally for approximately 30 minutes before being sacrificed. Bone marrow cells were then collected by flushing 1 ml of PBS from femurs. Cultured bone marrow cells and primary MEFs were incubated with 0.1 µg/mL colcemid for 2–6 hours at 37°C to allow mitotic cells to accumulate. Metaphase spreads were obtained by incubating mouse cells in 0.075 M KCl for 15 minutes at 37°C, followed by fixing cells in ice-cold (3∶1) methanol and glacial acetic acid and dropping the fixed cells onto slides. Metaphase spreads were hybridized with Cy3-labeled (CCCTAA)_3_ (0.3 µg/mL, Panagene), washed, and then counterstained with 4, 6 diamidino-2-phenylindole (DAPI) as previously described [56]. Images were captured using Cytovision software (Applied Imaging Corp.) on a fluorescence microscope (Axio2; Carl Zeiss, Germany); followed by quantification of individual telomere fluorescence signals using the TFL-Telo software (a kind gift from P. Lansdorp, Vancouver, BC). For histograms and box/jitter plots, data from different mice of each genotype were scored using R statistical package (http://www.r-project.org/) and Graphpad software. Metaphases from different mice of each genotype were scored for fragile telomeres (a chromatid with ≥2 telomere signals) and SFEs (chromosome ends without detectable telomere signals).

### Chromosome-Orientation FISH (CO-FISH)

CO-FISH was used to measure T-SCEs as described previously [44]. Briefly, bone marrow cells were cultured in medium with 3∶1 ratio of BrdU/BrdC at a final concentration of 1×10^−5^ M for approximately 12 hours. Colcemid (0.1 µg/ml) was added for the final 2 hours. Metaphase spreads were prepared as described above, stained with Hoechst 33258, exposed to UV light and then digested with exonuclease III to remove newly synthesized strands. Remaining parental strands were hybridized with Cy3-labeled (CCCTAA)_3_ probe, then briefly washed with hybridization buffer and subsequently with Alexa 488–labeled (TTAGGG)_3_ probes. Hybridization and wash conditions were identical to those described for telomeric FISH. A chromosome with more than two telomeric DNA signals by both probes was scored as T-SCE positive.

### IF and telomere FISH

Fibroblasts were grown overnight on chamber slides and bone marrow cells were spun onto Cytospin microscopic slides at 200 rpm for 3 minutes. Cells were washed in PBS, fixed in 2% paraformaldehyde for 10 minutes at room temperature, permeabilized with 0.5% tritonX-100 for 5 minutes on ice and blocked with 10% FBS for 1–2 hours. Cells were stained overnight at 4°C with a rabbit anti-γH2AX antibody (1∶200, Santa cruz) or a rabbit anti-53BP1 antibody (1∶500, Novus Biologicals), followed by Alexa 488-labeled secondary antibody (1∶500; Molecular Probes) for one hour at room temperature. Cells were washed in PBS, fixed in 2% paraformaldehyde for 10 minutes at room temperature, washed, dehydrated through ethanol series and briefly dried. Slides were immediately hybridized to Cy3-labeled (CCCTAA)_3_ probe (Panagene) for 2 hours at room temperature. Hybridization buffer and wash conditions were identical to those described for telomeric FISH. Slides were counterstained with DAPI. Z-stack images were captured on a fluorescence microscope (Axiovert 200M; Carl Zeiss).

### Apoptosis assay

Apoptosis was quantified using the Annexin V: FITC Apoptosis Detection Kit as per the manufacturer's instructions (BD Pharmingen). Briefly, cells were harvested, washed twice with ice-cold PBS and resuspended in 1× Binding buffer supplied by the manufacturer. Cells were incubated with 5 µL PI and 5 µL FITC-Annexin V at room temperature for 15 minutes and analyzed using an Accuri O6 flow cytometer with FlowJo software.

### Western blot

Cells were exposed to 5 µM aphidicolin for 8 hours or 2 mM HU for 24 hours as a positive control for Chk1 phosphorylation or 10 Gy Ionizing radiation with 1 hour recovery as a positive control for Chk2 phosphorylation [37]. Cells were lysed with lysis buffer (50 mM Tris HCl, pH 7.4, 150 mM NaCl, 1% IGEPAL, 0.25% sodium deoxycholate, 0.5 M EDTA, 20% SDS) supplemented with complete, EDTA-free Protease Inhibitor Cocktail Tablet and PhosSTOP Phosphatase Inhibitor Cocktail Tablet (Roche). Approximately 40–100 µg cell extracts were examined for phosphorylated-Chk1 or Chk2 and actin by rabbit phospho-Chk1 (S345) (1: 1000, Cell signaling, 2341), mouse Anti-Chk2 (1∶500, BD Biosciences, 611570) or actin (1∶1000, Santa Cruz, SC-1616) antibodies.

## Supporting Information

Figure S1 Oxidative base lesion detection by the quantitative telomere-PCR method. (A) A standard curve for Endonuclease III-sensitive DNA lesions at telomere repeats. Double stranded telomere sequence containing oligonucleotides with various numbers of Tg lesions are digested with Endonuclease III. A ΔC_t_ is calculated based on the amplification profiles of Endonuclease III-treated and the mock-treated oligonucleotides. The numbers of Endonuclease III-sensitive lesions are calculated based on the equation of the regression line. (B) Quantitative telomere-PCR standard curves of mock- and Endonuclease III-treated synthetic telomere oligonucleotides. 84-oligomers contain either zero (Tg-free) or 1, 2, 4, or 8 Tg lesions (Tg-containing) (see Table S1). (C) Quantitative telomere-PCR standard curves of mock- and Endonuclease III-treated genomic DNA from wild-type and *Nth1^−/−^* mouse kidney.
(TIF)

Figure S2 Detection of oxidative base lesions in telomeric and non-telomeric loci. Endonuclease III-sensitive DNA lesions at the *36B4* or telomeric locus in wild-type and *Nth1^−/−^* MEFs and kidney tissue. Fold change is obtained by normalizing the ΔC_t_ values in a sample to that of wild-type control (the value was set to 1).
(TIF)

Figure S3 Fragile telomeres in wild-type and *Ogg1^−/−^* mouse cells. Metaphase spreads are analyzed by telomere-FISH. (A) Percentage of fragile telomeres in freshly isolated bone marrow cells (4 mice). (B) Percentage of fragile telomeres in stimulated bone marrow cells in culture (4 mice). Error bars indicate standard deviation. Student's *t*-test is used for statistical analysis. P-values are indicated.
(TIF)

Figure S4 DNA damage foci in wild-type and *Nth1^−/−^* bone marrow cells. IF and IF-telomere FISH analysis of *ex vivo* stimulated bone marrow. (A) Percentage of wild-type and *Nth1^−/−^* cells with various numbers of γH2AX foci. (B) Percentage of wild-type and *Nth1^−/−^* cells with greater than or equal to three γH2AX foci that colocalize with telomere DNA.
(TIF)

Figure S5 Chk1 and Chk2 phosphorylation in wild-type and *Nth1^−/−^* primary MEFs. (A–B) Representative western blot analysis for Chk1 phosphorylation. For a positive control, cells are exposed to 5 µM APH for 8 hours (A) or 2 mM HU for 24 hours (B). (C) Representative western blot analysis for Chk2 phosphorylation. For a positive control, cells are exposed to 10 Gy Ionizing radiation (IR) and recovered for one hour. Actin serves as a loading control.
(TIF)

Figure S6 Telomere length in wild-type and *Nth1^−/−^* mouse tissues. (A) Flow-FISH analysis of freshly isolated splenocytes (24 mice) and bone marrow cells (16 mice). (B) A representative jitter plot (left panel) and a combined histogram (right panel) of telomere signal intensity by Q-FISH analysis from metaphase spreads of freshly isolated bone marrow cells with indicated genotype (n = 10 mice). Bars (in green) denote mean telomere signal intensity.
(TIF)

Figure S7 DNA damage response and cell apoptosis in wild-type and *Nth1^−/−^* mouse cells with or without telomerase. (A) Representative western blot analysis for Chk1 and Chk2 phosphorylation in mouse cells with indicated genotype. Actin serves as a loading control. (B–C) Percent apoptotic cells in *Nth1^+/+^ Tert^−/−^* and *Nth1^−/−^Tert^−/−^* mice (n = 4) and in wild-type and *Nth1^−/−^* mice (n = 6). Bone marrow cells are stimulated in culture with or without exposure to 2 mM HU for 24 hours or with 0.2 and 1 µM aphidicolin for 16 hours, released and analyzed at the indicated time points. Cells are stained with FITC-AnnexinV. Error bars denote standard error of mean (SEM). P-values are calculated using a Student's *t*-test and adjusted using Benjamini-Hochberg False Discovery Rate -controlling method [57]. P-values<0.05 are statistically significant using the above method.
(TIF)

Table S1 Sequences of oligonucleotides used as template to construct standard curves.
(DOCX)

Table S2 qPCR efficiencies and correlation coefficients for the Tg containing oligomers and mouse DNA.
(DOCX)

## References

[1] de Lange T (2005) Shelterin: the protein complex that shapes and safeguards human telomeres. Genes Dev 19: 2100–2110. 16166375 10.1101/gad.1346005

[2] d'Adda di Fagagna F, Teo SH, Jackson SP (2004) Functional links between telomeres and proteins of the DNA-damage response. Genes Dev 18: 1781–1799. 15289453 10.1101/gad.1214504

[3] Blackburn EH (2001) Switching and signaling at the telomere. Cell 106: 661–673. 11572773 10.1016/s0092-8674(01)00492-5

[4] von Zglinicki T (2000) Role of oxidative stress in telomere length regulation and replicative senescence. Ann N Y Acad Sci 908: 99–110. 10911951 10.1111/j.1749-6632.2000.tb06639.x

[5] Liu Y, Harrington L (2012) Murine models of dysfunctional telomeres and telomerase; Lue N, Autexier C, editors. New Jersey: John Wiley & Sons, Inc. 213–242 p.

[6] Casper AM, Nghiem P, Arlt MF, Glover TW (2002) ATR regulates fragile site stability. Cell 111: 779–789. 12526805 10.1016/s0092-8674(02)01113-3

[7] Sfeir A, Kosiyatrakul ST, Hockemeyer D, MacRae SL, Karlseder J, et al (2009) Mammalian telomeres resemble fragile sites and require TRF1 for efficient replication. Cell 138: 90–103. 19596237 10.1016/j.cell.2009.06.021 PMC2723738

[8] Petersen S, Saretzki G, von Zglinicki T (1998) Preferential accumulation of single-stranded regions in telomeres of human fibroblasts. Exp Cell Res 239: 152–160. 9511733 10.1006/excr.1997.3893

[9] Rhee DB, Ghosh A, Lu J, Bohr VA, Liu Y (2010) Factors that influence telomeric oxidative base damage and repair by DNA glycosylase OGG1. DNA Repair (Amst) 10: 34–44. 20951653 10.1016/j.dnarep.2010.09.008 PMC3010491

[10] Rochette PJ, Brash DE (2010) Human telomeres are hypersensitive to UV-induced DNA Damage and refractory to repair. PLoS Genet 6: e1000926. 20442874 10.1371/journal.pgen.1000926 PMC2861706

[11] Fumagalli M, Rossiello F, Clerici M, Barozzi S, Cittaro D, et al (2012) Telomeric DNA damage is irreparable and causes persistent DNA-damage-response activation. Nat Cell Biol 14: 355–365. 22426077 10.1038/ncb2466 PMC3717580

[12] Hewitt G, Jurk D, Marques FD, Correia-Melo C, Hardy T, et al (2012) Telomeres are favoured targets of a persistent DNA damage response in ageing and stress-induced senescence. Nat Commun 3: 708–717. 22426229 10.1038/ncomms1708 PMC3292717

[13] Henle ES, Han Z, Tang N, Rai P, Luo Y, et al (1999) Sequence-specific DNA cleavage by Fe2+-mediated fenton reactions has possible biological implications. J Biol Chem 274: 962–971. 9873038 10.1074/jbc.274.2.962

[14] Oikawa S, Kawanishi S (1999) Site-specific DNA damage at GGG sequence by oxidative stress may accelerate telomere shortening. FEBS Lett 453: 365–368. 10405177 10.1016/s0014-5793(99)00748-6

[15] Oikawa S, Tada-Oikawa S, Kawanishi S (2001) Site-specific DNA damage at the GGG sequence by UVA involves acceleration of telomere shortening. Biochemistry 40: 4763–4768. 11294644 10.1021/bi002721g

[16] David SS, O'Shea VL, Kundu S (2007) Base-excision repair of oxidative DNA damage. Nature 447: 941–950. 17581577 10.1038/nature05978 PMC2896554

[17] Svilar D, Goellner EM, Almeida KH, Sobol RW (2011) Base excision repair and lesion-dependent subpathways for repair of oxidative DNA damage. Antioxid Redox Signal 14: 2491–2507. 20649466 10.1089/ars.2010.3466 PMC3096496

[18] Clark JM, Beardsley GP (1986) Thymlne glycol lesions terminate chain elongation by DNA polymerase I in vitro. Nucleic Acids Research 14: 737–749. 3511447 10.1093/nar/14.2.737 PMC339461

[19] Evans J, Maccabee M, Hatahet Z, Courcelle J, Bockrath R, et al (1993) Thymine ring saturation and fragmentation products: lesion bypass, misinsertion and implications for mutagenesis. Mutation Research/Genetic Toxicology 299: 147–156. 10.1016/0165-1218(93)90092-r 7683083

[20] Aller P, Rould MA, Hogg M, Wallace SS, Doublie S (2007) A structural rationale for stalling of a replicative DNA polymerase at the most common oxidative thymine lesion, thymine glycol. Proc Natl Acad Sci U S A 104: 814–818. 17210917 10.1073/pnas.0606648104 PMC1783396

[21] Opresko PL, Fan J, Danzy S, Wilson DM 3rd, Bohr VA (2005) Oxidative damage in telomeric DNA disrupts recognition by TRF1 and TRF2. Nucleic Acids Res 33: 1230–1239. 15731343 10.1093/nar/gki273 PMC549571

[22] Lu J, Liu Y (2010) Deletion of Ogg1 DNA glycosylase results in telomere base damage and length alteration in yeast. Embo J 29: 398–409. 19942858 10.1038/emboj.2009.355 PMC2824463

[23] Hegde ML, Hazra TK, Mitra S (2008) Early steps in the DNA base excision/single-strand interruption repair pathway in mammalian cells. Cell Res 18: 27–47. 18166975 10.1038/cr.2008.8 PMC2692221

[24] Hu J, de Souza-Pinto NC, Haraguchi K, Hogue BA, Jaruga P, et al (2005) Repair of Formamidopyrimidines in DNA Involves Different Glycosylases. Journal of Biological Chemistry 280: 40544–40551. 16221681 10.1074/jbc.M508772200

[25] Luna L, Bjørås M, Hoff E, Rognes T, Seeberg E (2000) Cell-cycle regulation, intracellular sorting and induced overexpression of the human NTH1 DNA glycosylase involved in removal of formamidopyrimidine residues from DNA. Mutation Research/DNA Repair 460: 95–104. 10.1016/s0921-8777(00)00015-x 10882850

[26] Wang Z, Rhee DB, Lu J, Bohr CT, Zhou F, et al (2010) Characterization of oxidative guanine damage and repair in mammalian telomeres. PLoS Genet 6: e1000951. 20485567 10.1371/journal.pgen.1000951 PMC2869316

[27] O'Callaghan N, Baack N, Sharif R, Fenech M (2012) A qPCR-based assay to quantify oxidized guanine and other FPG-sensitive base lesions within telomeric DNA. Biotechniques 51: 403–411. 10.2144/000113788 22150331

[28] Leadon SA, Stampfer MR, Bartley J (1988) Production of oxidative DNA damage during the metabolic activation of benzo[a]pyrene in human mammary epithelial cells correlates with cell killing. Proc Natl Acad Sci U S A 85: 4365–4368. 3380798 10.1073/pnas.85.12.4365 PMC280429

[29] Martinez P, Thanasoula M, Munoz P, Liao C, Tejera A, et al (2009) Increased telomere fragility and fusions resulting from TRF1 deficiency lead to degenerative pathologies and increased cancer in mice. Genes Dev 23: 2060–2075. 19679647 10.1101/gad.543509 PMC2751970

[30] Badie S, Escandell JM, Bouwman P, Carlos AR, Thanasoula M, et al (2010) BRCA2 acts as a RAD51 loader to facilitate telomere replication and capping. Nat Struct Mol Biol 17: 1461–1469. 21076401 10.1038/nsmb.1943 PMC2998174

[31] McNees CJ, Tejera AM, Martinez P, Murga M, Mulero F, et al (2010) ATR suppresses telomere fragility and recombination but is dispensable for elongation of short telomeres by telomerase. J Cell Biol 188: 639–652. 20212315 10.1083/jcb.200908136 PMC2835929

[32] Gu P, Min JN, Wang Y, Huang C, Peng T, et al (2012) CTC1 deletion results in defective telomere replication, leading to catastrophic telomere loss and stem cell exhaustion. Embo J 31: 2309–2321. 22531781 10.1038/emboj.2012.96 PMC3364752

[33] Remeseiro S, Cuadrado A, Carretero M, Martinez P, Drosopoulos WC, et al (2012) Cohesin-SA1 deficiency drives aneuploidy and tumourigenesis in mice due to impaired replication of telomeres. EMBO J 31: 2076–2089. 22415365 10.1038/emboj.2012.11 PMC3343459

[34] Sedelnikova OA, Redon CE, Dickey JS, Nakamura AJ, Georgakilas AG, et al (2010) Role of oxidatively induced DNA lesions in human pathogenesis. Mutation Research/Reviews in Mutation Research 704: 152–159. 10.1016/j.mrrev.2009.12.005 PMC3074954 20060490

[35] Ciccia A, Elledge SJ (2010) The DNA damage response: making it safe to play with knives. Mol Cell 40: 179–204. 20965415 10.1016/j.molcel.2010.09.019 PMC2988877

[36] Glover TW, Berger C, Coyle J, Echo B (1984) DNA polymerase alpha inhibition by aphidicolin induces gaps and breaks at common fragile sites in human chromosomes. Hum Genet 67: 136–142. 6430783 10.1007/BF00272988

[37] Brown EJ, Baltimore D (2003) Essential and dispensable roles of ATR in cell cycle arrest and genome maintenance. Genes Dev 17: 615–628. 12629044 10.1101/gad.1067403 PMC196009

[38] Harley CB, Futcher AB, Greider CW (1990) Telomeres shorten during ageing of human fibroblasts. Nature 345: 458–460. 2342578 10.1038/345458a0

[39] Hastie ND, Dempster M, Dunlop MG, Thompson AM, Green DK, et al (1990) Telomere reduction in human colorectal carcinoma and with ageing. Nature 346: 866–868. 2392154 10.1038/346866a0

[40] Wright WE, Piatyszek MA, Rainey WE, Byrd W, Shay JW (1996) Telomerase activity in human germline and embryonic tissues and cells. Dev Genet 18: 173–179. 8934879 10.1002/(SICI)1520-6408(1996)18:2<173::AID-DVG10>3.0.CO;2-3

[41] Prowse KR, Greider CW (1995) Developmental and tissue-specific regulation of mouse telomerase and telomere length. Proc Natl Acad Sci U S A 92: 4818–4822. 7761406 10.1073/pnas.92.11.4818 PMC41798

[42] Hemann MT, Strong MA, Hao LY, Greider CW (2001) The shortest telomere, not average telomere length, is critical for cell viability and chromosome stability. Cell 107: 67–77. 11595186 10.1016/s0092-8674(01)00504-9

[43] Lee HW, Blasco MA, Gottlieb GJ, Horner JW 2nd, Greider CW, et al (1998) Essential role of mouse telomerase in highly proliferative organs. Nature 392: 569–574. 9560153 10.1038/33345

[44] Bailey SM, Goodwin EH, Cornforth MN (2004) Strand-specific fluorescence in situ hybridization: the CO-FISH family. Cytogenet Genome Res 107: 14–17. 15305050 10.1159/000079565

[45] Hagelstrom RT, Blagoev KB, Niedernhofer LJ, Goodwin EH, Bailey SM (2010) Hyper telomere recombination accelerates replicative senescence and may promote premature aging. Proc Natl Acad Sci U S A 107: 15768–15773. 20798040 10.1073/pnas.1006338107 PMC2936608

[46] Chan MK, Ocampo-Hafalla MT, Vartanian V, Jaruga P, Kirkali G, et al (2009) Targeted deletion of the genes encoding NTH1 and NEIL1 DNA N-glycosylases reveals the existence of novel carcinogenic oxidative damage to DNA. DNA Repair 8: 786–794. 19346169 10.1016/j.dnarep.2009.03.001 PMC4894318

[47] Karahalil B, de Souza-Pinto NC, Parsons JL, Elder RH, Bohr VA (2003) Compromised incision of oxidized pyrimidines in liver mitochondria of mice deficient in NTH1 and OGG1 glycosylases. J Biol Chem 278: 33701–33707. 12819227 10.1074/jbc.M301617200

[48] Otterlei M, Warbrick E, Nagelhus TA, Haug T, Slupphaug G, et al (1999) Post-replicative base excision repair in replication foci. Embo J 18: 3834–3844. 10393198 10.1093/emboj/18.13.3834 PMC1171460

[49] Oyama M, Wakasugi M, Hama T, Hashidume H, Iwakami Y, et al (2004) Human NTH1 physically interacts with p53 and proliferating cell nuclear antigen. Biochem Biophys Res Commun 321: 183–191. 15358233 10.1016/j.bbrc.2004.06.136

[50] de Souza-Pinto NC, Maynard S, Hashiguchi K, Hu J, Muftuoglu M, et al (2009) The recombination protein RAD52 cooperates with the excision repair protein OGG1 for the repair of oxidative lesions in mammalian cells. Mol Cell Biol 29: 4441–4454. 19506022 10.1128/MCB.00265-09 PMC2725742

[51] Maynard S, Schurman SH, Harboe C, de Souza-Pinto NC, Bohr VA (2009) Base excision repair of oxidative DNA damage and association with cancer and aging. Carcinogenesis 30: 2–10. 18978338 10.1093/carcin/bgn250 PMC2639036

[52] Liu Y, Snow BE, Hande MP, Yeung D, Erdmann NJ, et al (2000) The telomerase reverse transcriptase is limiting and necessary for telomerase function in vivo. Curr Biol 10: 1459–1462. 11102810 10.1016/s0960-9822(00)00805-8

[53] Elder RH, Dianov GL (2002) Repair of dihydrouracil supported by base excision repair in mNTH1 knock-out cell extracts. J Biol Chem 277: 50487–50490. 12401779 10.1074/jbc.M208153200

[54] O'Callaghan N, Dhillon V, Thomas P, Fenech M (2008) A quantitative real-time PCR method for absolute telomere length. Biotechniques 44: 807–809. 18476834 10.2144/000112761

[55] Rufer N, Dragowska W, Thornbury G, Roosnek E, Lansdorp PM (1998) Telomere length dynamics in human lymphocyte subpopulations measured by flow cytometry. Nat Biotechnol 16: 743–747. 9702772 10.1038/nbt0898-743

[56] Zijlmans JM, Martens UM, Poon SS, Raap AK, Tanke HJ, et al (1997) Telomeres in the mouse have large inter-chromosomal variations in the number of T2AG3 repeats. Proc Natl Acad Sci U S A 94: 7423–7428. 9207107 10.1073/pnas.94.14.7423 PMC23837

[57] Benjamini Y, Hochberg Y (1995) Controlling the False Discovery Rate: A Practical and Powerful Approach to Multiple Testing. J R Statist Soc B 57: 289–300.

