# Supplementary figures and images for: Defective Repair of Oxidative Base Lesions by the DNA Glycosylase Nth1 Associates with Multiple Telomere Defects
Source: PLoS Genet. 2013 Jul 18;9(7):e1003639. doi: 10.1371/journal.pgen.1003639 (PMC3715427; doi:10.1371/journal.pgen.1003639)

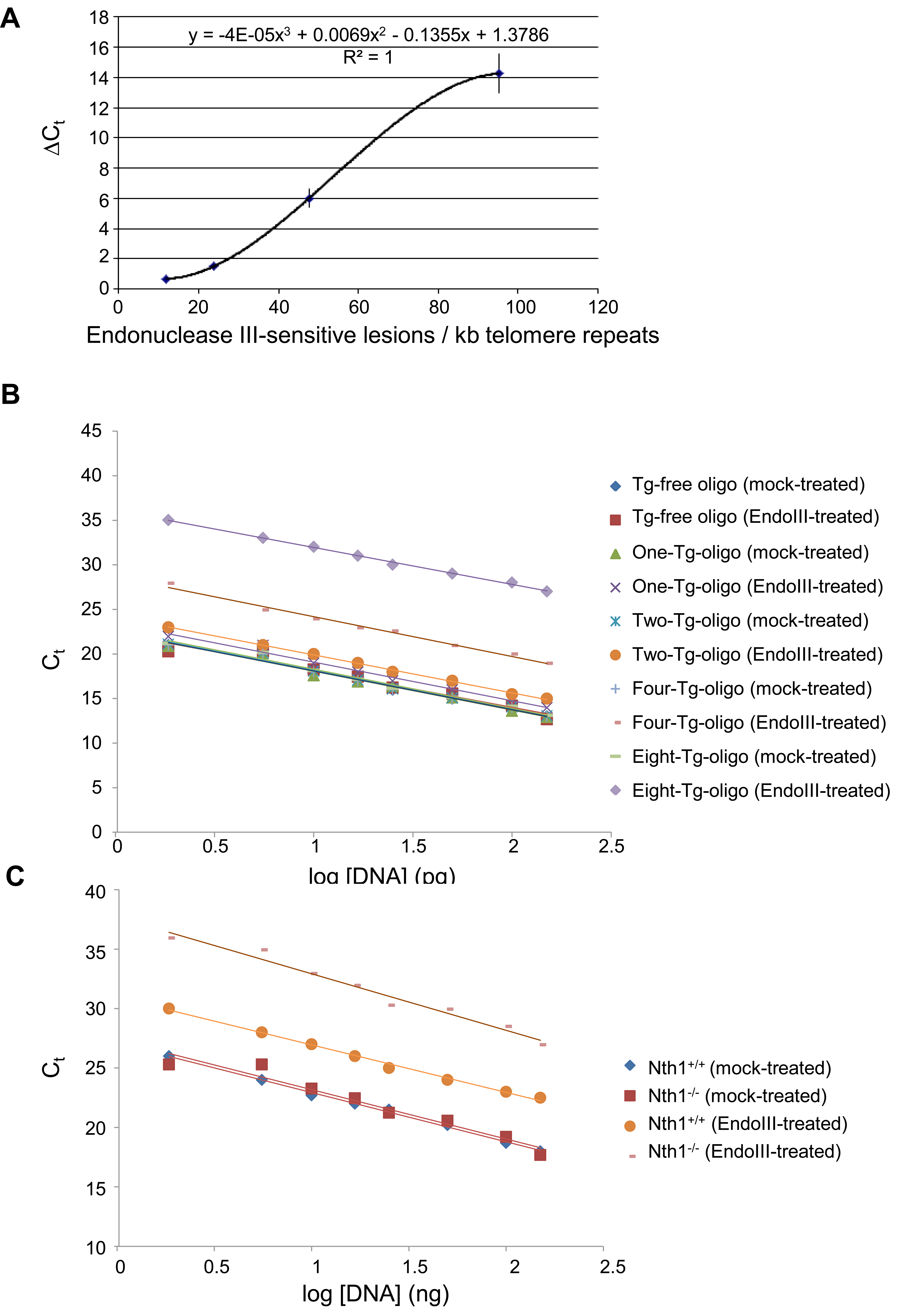

Supplement: Figure S1 — Oxidative base lesion detection by the quantitative telomere-PCR method. (A) A standard curve for Endonuclease III-sensitive DNA lesions at telomere repeats. Double stranded telomere sequence containing oligonucleotides with various numbers of Tg lesions are digested with Endonuclease III. A ΔCt is calculated based on the amplification profiles of Endonuclease III-treated and the mock-treated oligonucleotides. The numbers of Endonuclease III-sensitive lesions are calculated based on the equation of the regression line. (B) Quantitative telomere-PCR standard curves of mock- and Endonuclease III-treated synthetic telomere oligonucleotides. 84-oligomers contain either zero (Tg-free) or 1, 2, 4, or 8 Tg lesions (Tg-containing) (see Table S1). (C) Quantitative telomere-PCR standard curves of mock- and Endonuclease III-treated genomic DNA from wild-type and Nth1−/− mouse kidney. (TIF) [file pgen.1003639.s001.tif]

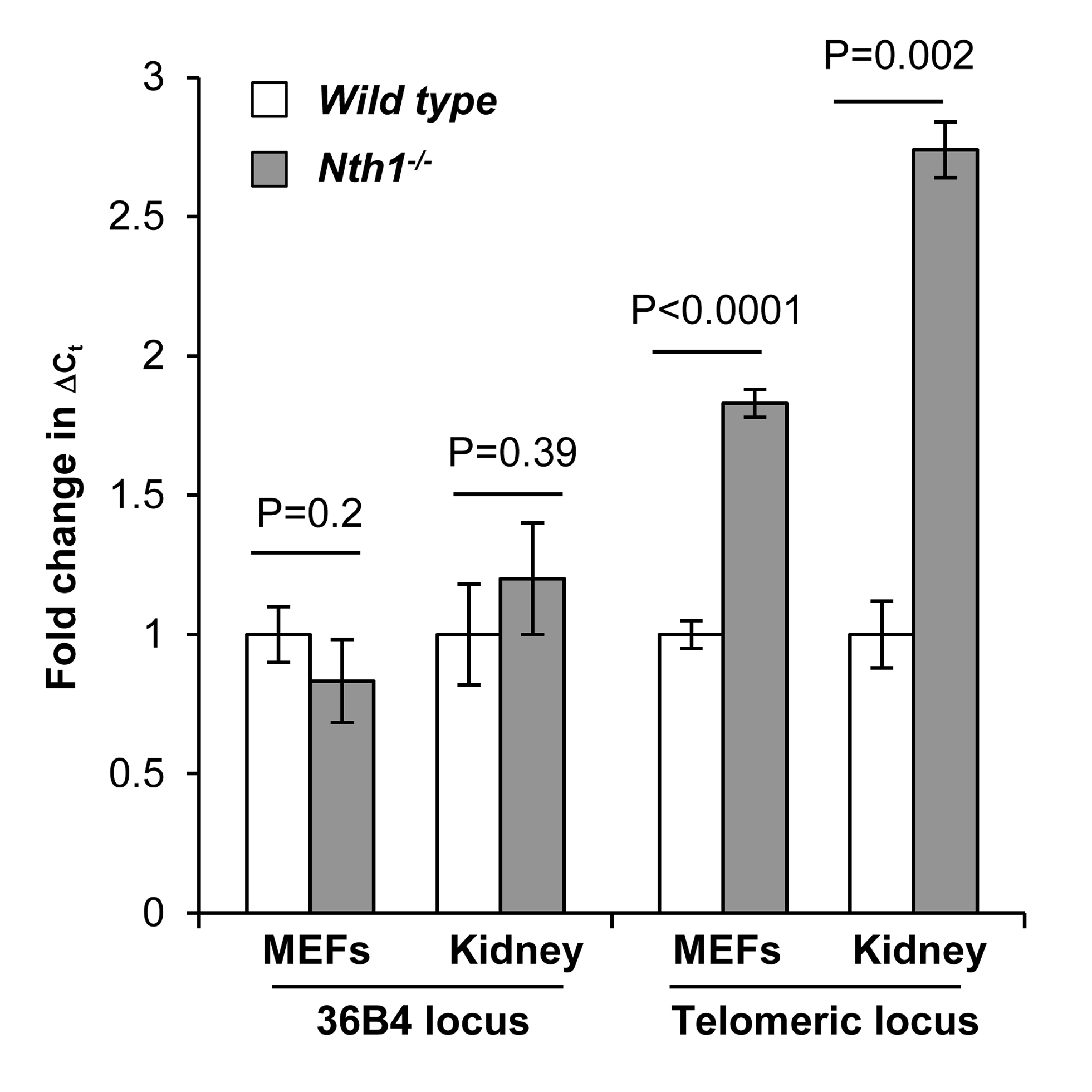

Supplement: Figure S2 — Detection of oxidative base lesions in telomeric and non-telomeric loci. Endonuclease III-sensitive DNA lesions at the 36B4 or telomeric locus in wild-type and Nth1−/− MEFs and kidney tissue. Fold change is obtained by normalizing the ΔCt values in a sample to that of wild-type control (the value was set to 1). (TIF) [file pgen.1003639.s002.tif]

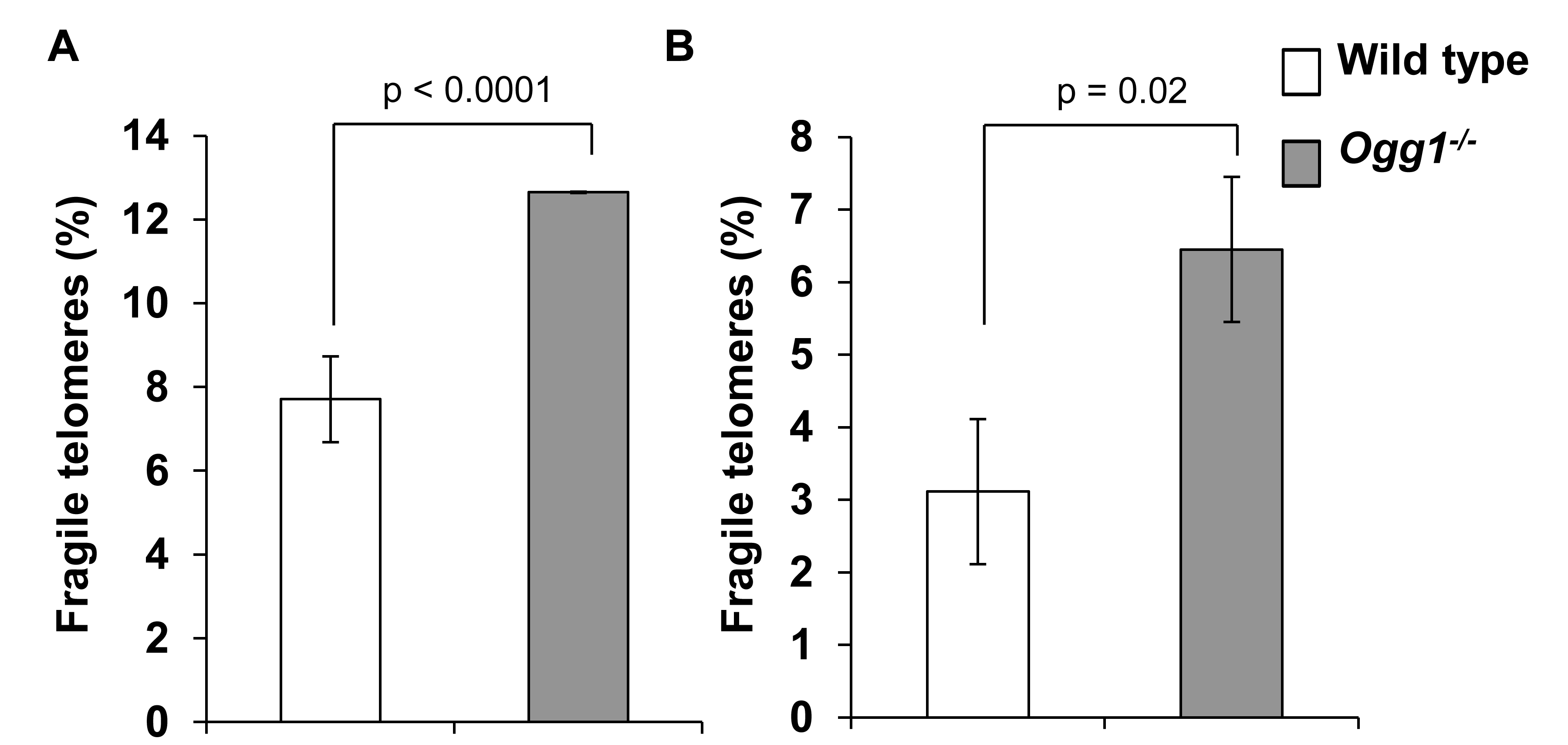

Supplement: Figure S3 — Fragile telomeres in wild-type and Ogg1−/− mouse cells. Metaphase spreads are analyzed by telomere-FISH. (A) Percentage of fragile telomeres in freshly isolated bone marrow cells (4 mice). (B) Percentage of fragile telomeres in stimulated bone marrow cells in culture (4 mice). Error bars indicate standard deviation. Student's t-test is used for statistical analysis. P-values are indicated. (TIF) [file pgen.1003639.s003.tif]

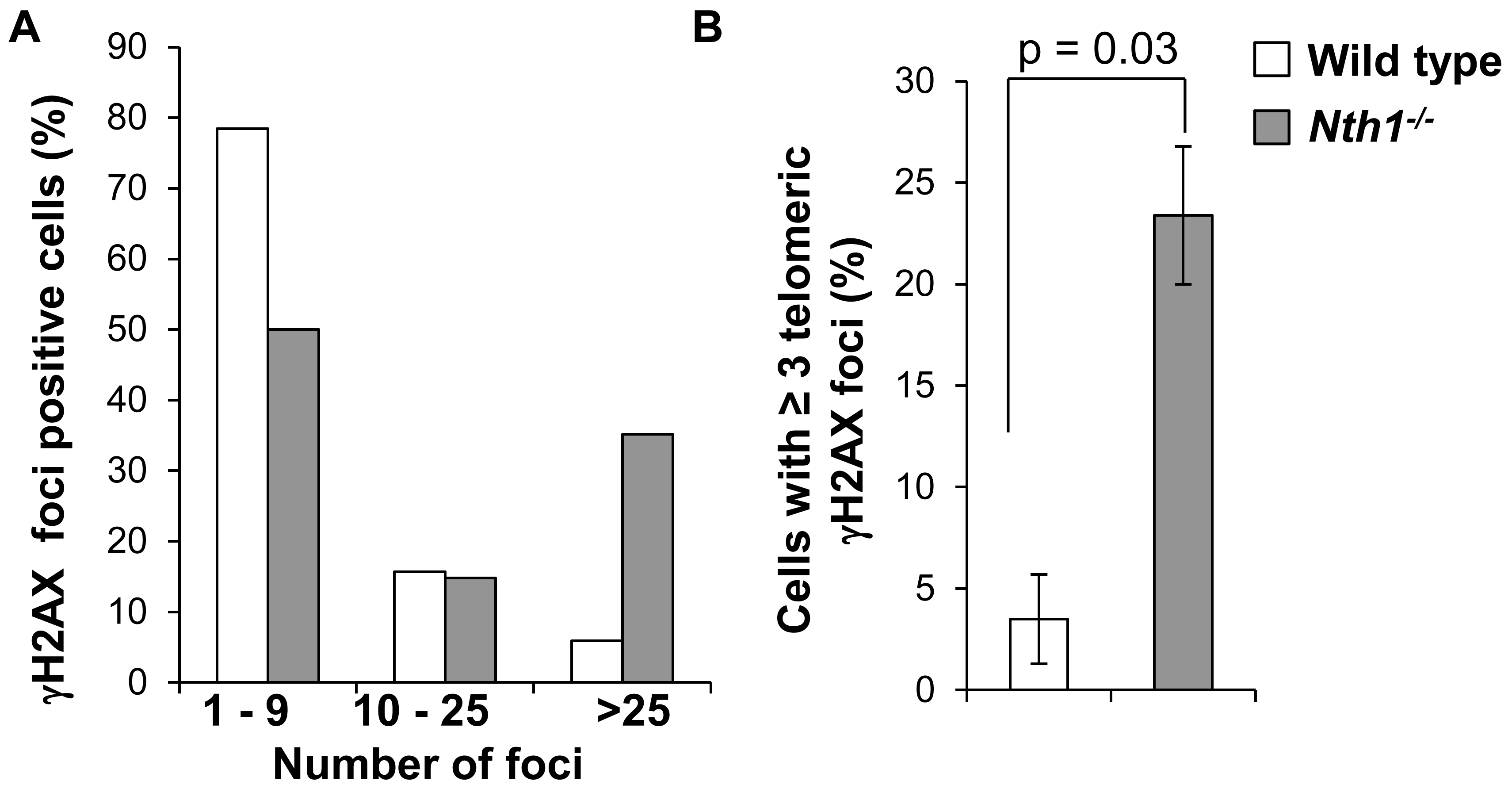

Supplement: Figure S4 — DNA damage foci in wild-type and Nth1−/− bone marrow cells. IF and IF-telomere FISH analysis of ex vivo stimulated bone marrow. (A) Percentage of wild-type and Nth1−/− cells with various numbers of γH2AX foci. (B) Percentage of wild-type and Nth1−/− cells with greater than or equal to three γH2AX foci that colocalize with telomere DNA. (TIF) [file pgen.1003639.s004.tif]

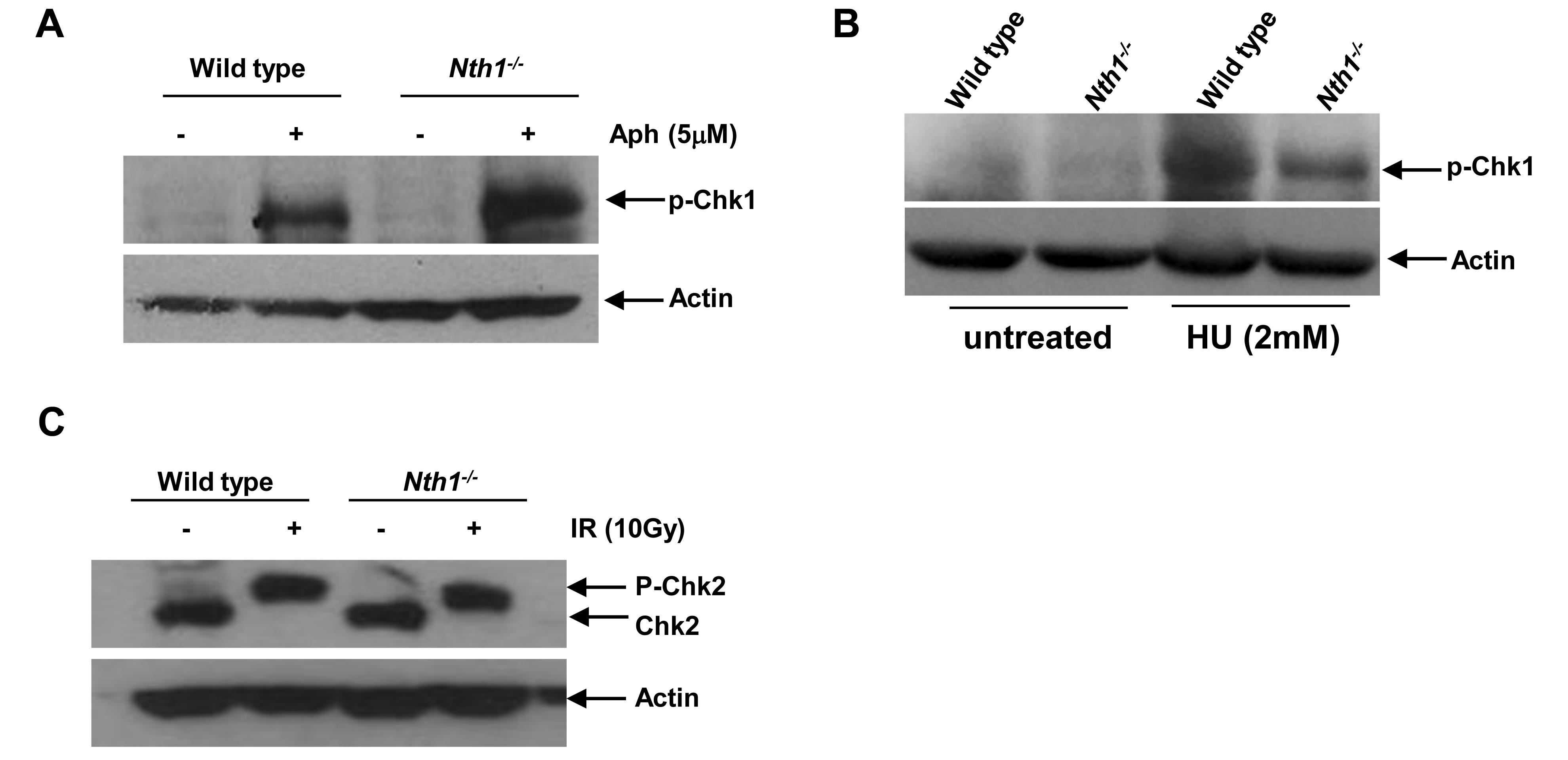

Supplement: Figure S5 — Chk1 and Chk2 phosphorylation in wild-type and Nth1−/− primary MEFs. (A–B) Representative western blot analysis for Chk1 phosphorylation. For a positive control, cells are exposed to 5 µM APH for 8 hours (A) or 2 mM HU for 24 hours (B). (C) Representative western blot analysis for Chk2 phosphorylation. For a positive control, cells are exposed to 10 Gy Ionizing radiation (IR) and recovered for one hour. Actin serves as a loading control. (TIF) [file pgen.1003639.s005.tif]

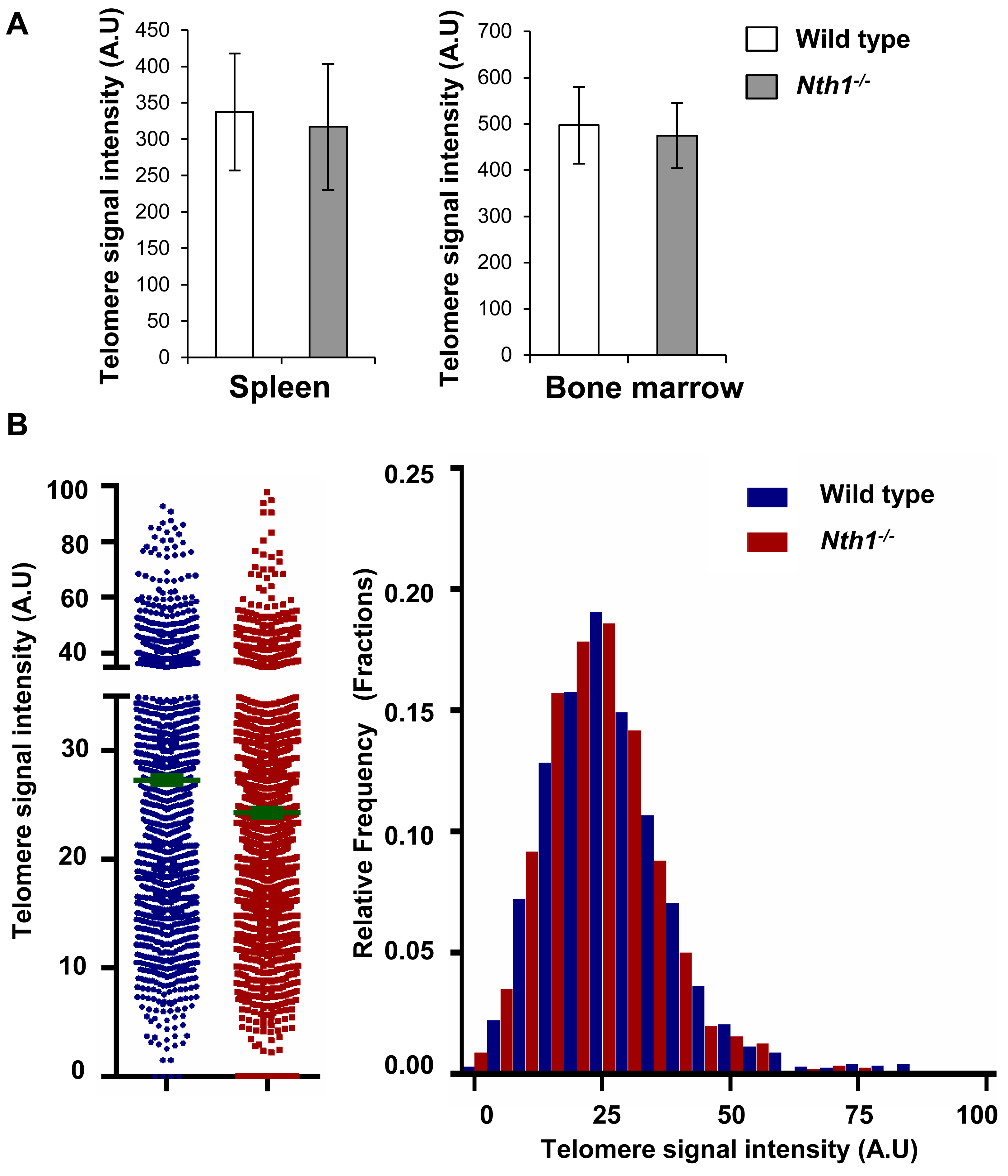

Supplement: Figure S6 — Telomere length in wild-type and Nth1−/− mouse tissues. (A) Flow-FISH analysis of freshly isolated splenocytes (24 mice) and bone marrow cells (16 mice). (B) A representative jitter plot (left panel) and a combined histogram (right panel) of telomere signal intensity by Q-FISH analysis from metaphase spreads of freshly isolated bone marrow cells with indicated genotype (n = 10 mice). Bars (in green) denote mean telomere signal intensity. (TIF) [file pgen.1003639.s006.tif]

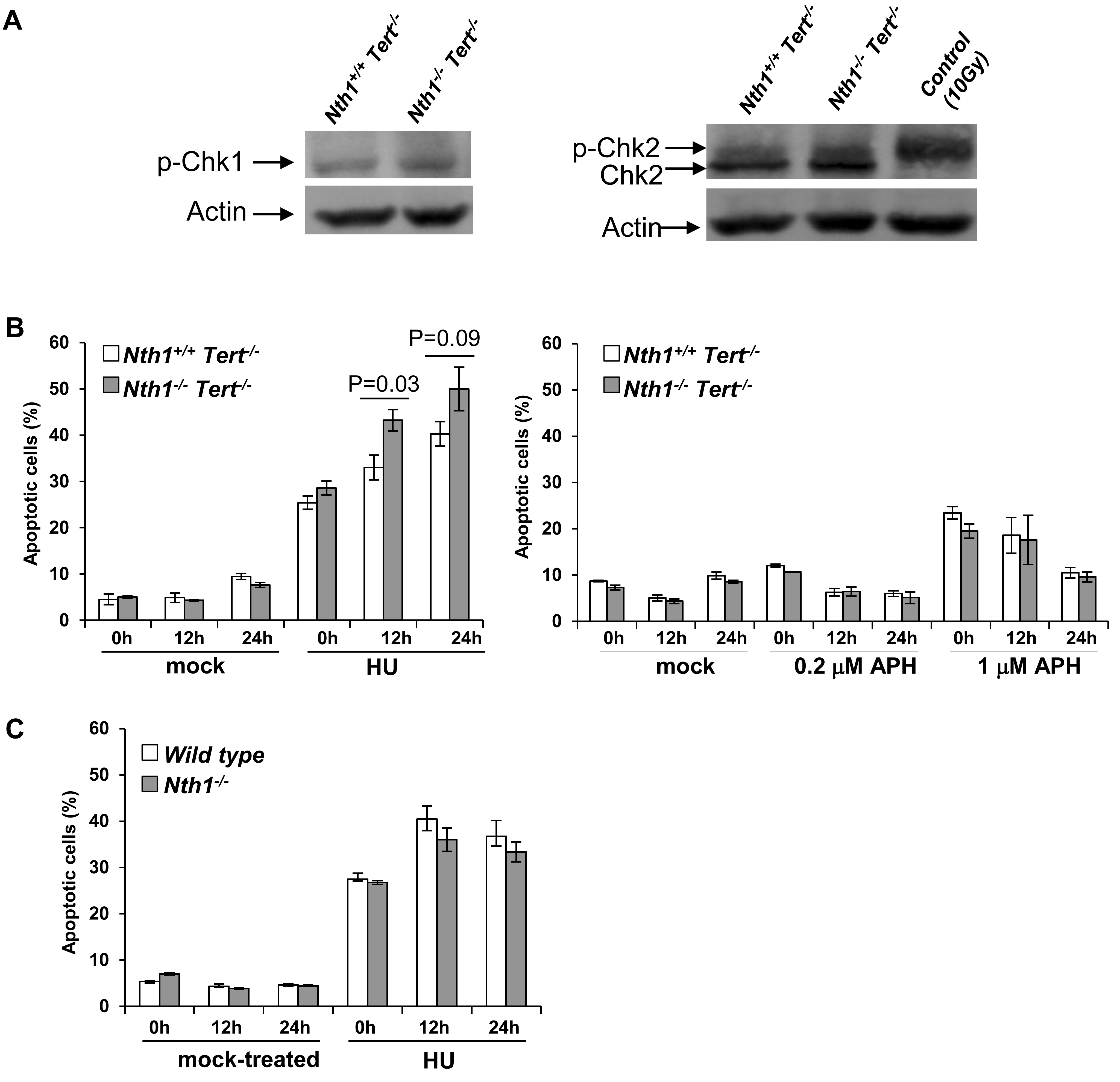

Supplement: Figure S7 — DNA damage response and cell apoptosis in wild-type and Nth1−/− mouse cells with or without telomerase. (A) Representative western blot analysis for Chk1 and Chk2 phosphorylation in mouse cells with indicated genotype. Actin serves as a loading control. (B–C) Percent apoptotic cells in Nth1+/+ Tert−/− and Nth1−/−Tert−/− mice (n = 4) and in wild-type and Nth1−/− mice (n = 6). Bone marrow cells are stimulated in culture with or without exposure to 2 mM HU for 24 hours or with 0.2 and 1 µM aphidicolin for 16 hours, released and analyzed at the indicated time points. Cells are stained with FITC-AnnexinV. Error bars denote standard error of mean (SEM). P-values are calculated using a Student's t-test and adjusted using Benjamini-Hochberg False Discovery Rate -controlling method [57]. P-values<0.05 are statistically significant using the above method. (TIF) [file pgen.1003639.s007.tif]
