# Supplementary material for: Defective Repair of Oxidative Base Lesions by the DNA Glycosylase Nth1 Associates with Multiple Telomere Defects
Source: PLoS Genet. 2013 Jul 18;9(7):e1003639. doi: 10.1371/journal.pgen.1003639 (PMC3715427; doi:10.1371/journal.pgen.1003639)
Supplement: Table S1 — Sequences of oligonucleotides used as template to construct standard curves. (DOCX) [file pgen.1003639.s008.docx]

Table S1. Sequences of oligonucleotides used as template to construct standard curves

| **Numbers of Tgs** | **EndoIII-sensitive sites**  **/kb telomere** | **Oligomer sequence (84 bases)** |
| --- | --- | --- |
| 0 | 0 | TTAGGGTTAGGGTTAGGGTTAGGGTTAGGGTTAGGGTTAGGGTTAGGGTTAGGGTTAGGGTTAGGGTTAGGGTTAGGGTTAGGG |
| 1 | 11.9 | TTAGGGTTAGGGTTAGGGTTAGGGTTAGGGTTAGGG***X***TAGGGTTAGGGTTAGGGTTAGGGTTAGGGTTAGGGTTAGGGTTAGGG |
| 2 | 23.8 | TTAGGGTTAGGGTTAGGG***X***TAGGGTTAGGGTTAGGGTTAGGGTTAGGGTTAGGG***X***TAGGGTTAGGGTTAGGGTTAGGGTTAGGG |
| 4 | 47.6 | TTAGGGTTAGGGTTAGGG***X***TAGGGTTAGGGTTAGGG***X***TAGGGTTAGGGTTAGGG***X***TAGGGTTAGGGTTAGGG***X***TAGGGTTAGGG |
| 8 | 95.2 | TTAGGGTTAGGG***X***TAGGG***X***TAGGG***X***TAGGGTTAGGG***X***TAGGG***X***TAGGGTTAGGG***X***TAGGGTTAGGG***X***TAGGG***X***TAGGGTTAGGG |
| Reverse | 0 | CCCTAACCCTAACCCTAACCCTAACCCTAACCCTAACCCTAACCCTAACCCTAACCCTAACCCTAACCCTAACCCTAACCCTAA |

*X* denotes Tg lesions
