# Supplementary material for: Defective Repair of Oxidative Base Lesions by the DNA Glycosylase Nth1 Associates with Multiple Telomere Defects
Source: PLoS Genet. 2013 Jul 18;9(7):e1003639. doi: 10.1371/journal.pgen.1003639 (PMC3715427; doi:10.1371/journal.pgen.1003639)
Supplement: Table S2 — qPCR efficiencies and correlation coefficients for the Tg containing oligomers and mouse DNA. (DOCX) [file pgen.1003639.s009.docx]

Table S2. qPCR efficiencies and correlation coefficients for the Tg containing oligomers and mouse DNA

| Number of Tg in the 84 base oligomers | EndoIII treated | Slope | Exponent | Amplification | Efficiency (%) | Correlation  Coefficient |
| --- | --- | --- | --- | --- | --- | --- |
| 0 | No | -4.1 | 0.244 | 1.75 | 75.349 | 0.997 |
|  | Yes | -4.15 | 0.241 | 1.742 | 74.166 | 0.998 |
| 1 | No | -4.18 | 0.239 | 1.735 | 73.474 | 0.993 |
|  | Yes | -4.20 | 0.231 | 1.73 | 73.02 | 0.992 |
| 2 | No | -4.12 | 0.243 | 1.749 | 74.871 | 0.9915 |
|  | Yes | -4.15 | 0.241 | 1.742 | 74.166 | 0.9924 |
| 4 | No | -4.20 | 0.231 | 1.73 | 73.02 | 0.993 |
|  | Yes | -4.22 | 0.237 | 1.726 | 72.571 | 0.9929 |
| 8 | No | -4.19 | 0.239 | 1.732 | 73.246 | 0.9901 |
|  | Yes | -4.23 | 0.236 | 1.723 | 72.348 | 0.9913 |
| Genomic DNA | No | -3.989 | 0.251 | 1.781 | 78.11 | 0.9978 |
|  | Yes | -4.015 | 0.249 | 1.774 | 77.446 | 0.9981 |

An example of the standard curves is given in Figure S1. The formula for this calculation is

Efficiency = 10^(-1/slope)^ -1
